# ALS Associated Mutations in Matrin 3 Alter Protein-Protein Interactions and Impede mRNA Nuclear Export

**DOI:** 10.1038/s41598-017-14924-6

**Published:** 2017-11-06

**Authors:** Ashley Boehringer, Krystine Garcia-Mansfield, Gurkaran Singh, Nadine Bakkar, Patrick Pirrotte, Robert Bowser

**Affiliations:** 1 Department of Neurobiology, Barrow Neurological Institute, Phoenix, AZ USA; 2 School of Life Sciences, Arizona State University, Phoenix, AZ USA; 3 Collaborative Center for Translational Mass Spectrometry, Translational Genomics Research Institute, Phoenix, AZ USA

## Abstract

Mutations in Matrin 3 have recently been linked to ALS, though the mechanism that induces disease in these patients is unknown. To define the protein interactome of wild-type and ALS-linked *MATR3* mutations, we performed immunoprecipitation followed by mass spectrometry using NSC-34 cells expressing human wild-type or mutant Matrin 3. Gene ontology analysis identified a novel role for Matrin 3 in mRNA transport centered on proteins in the TRanscription and EXport (TREX) complex, known to function in mRNA biogenesis and nuclear export. ALS-linked mutations in Matrin 3 led to its re-distribution within the nucleus, decreased co-localization with endogenous Matrin 3 and increased co-localization with specific TREX components. Expression of disease-causing Matrin 3 mutations led to nuclear mRNA export defects of both global mRNA and more specifically the mRNA of TDP-43 and FUS. Our findings identify a potential pathogenic mechanism attributable to *MATR3* mutations and further link cellular transport defects to ALS.

## Introduction

Amyotrophic lateral sclerosis (ALS) is a progressive neurodegenerative disorder that results in the loss of motor neurons in the brain, brain stem, and spinal cord^1,2^. Loss of motor neurons results in muscle atrophy and progressive paralysis, typically leading to death due to respiratory failure within 2–5 years of diagnosis. Amongst a growing number of genetic mutations linked to ALS, the most common genetic cause of ALS is a repeat expansion of the *C9orf72* locus^3,4^. Of the more than 30 genes associated with ALS^5^, the most common mechanistic pathway implicated in ALS is RNA processing and metabolism. Mutations in many proteins that function in RNA processing and regulation such as TDP-43^6,7^, FUS^8^, hnRNPA1, hnRNPA2B1^9^ and Matrin 3^10^ have been linked to ALS. However, the manner in which defects in RNA processing lead to neurodegeneration remains poorly understood.

**Figure 1:**
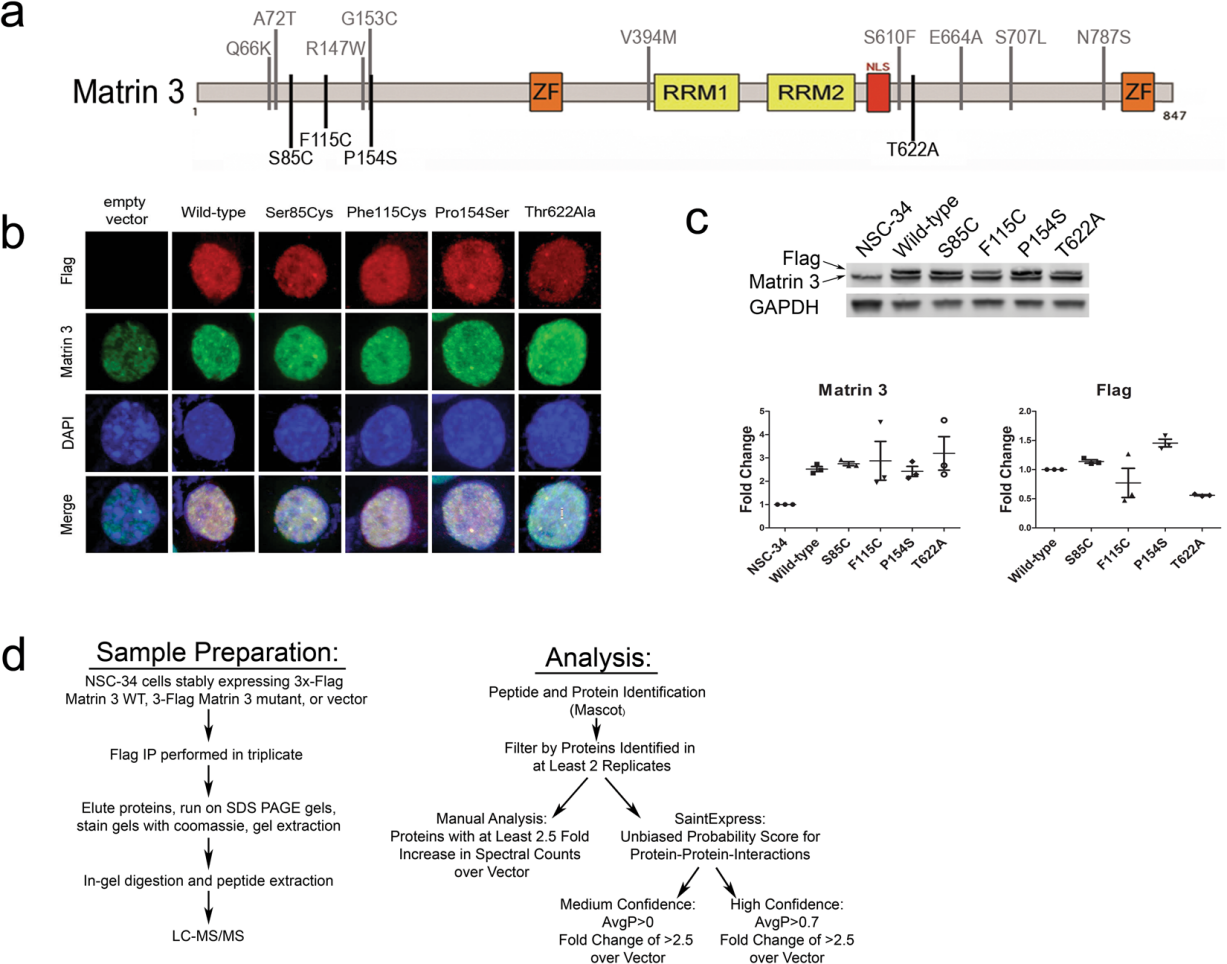
Matrin 3 cell culture model and IP-MS workflow. (**a**) Domain structure of Matrin 3 including location of mutants studied in this work listed below the protein as well as other recently identified mutations shown above. (**b**) Representative immunofluorescence images of NSC-34 cells stably expressing Flag tagged wild-type Matrin 3 or one of S85C, F115C, P154S or T622A Matrin 3 mutants. Cells transfected with empty vector are also shown denoting endogenous levels and localization of Matrin 3. Flag expression is shown in red, Matrin 3 in green, DAPI marking nucleus in blue. (**c**) Western blot of whole cell lysates probed with antibodies against Flag (top) and Matrin 3 (bottom) showing expression levels of endogenous Matrin 3 and Flag tagged Matrin 3 in NSC-34 stable cells lines and quantitation. Error bars represent standard error of the mean (SEM) of three experiments. One way Analysis of Variance (ANOVA) with Dunnett’s post-test showed no significant differences between level of expression of wild-type and any of the four mutations (Flag *p-*values: WT vs 85: *p* = 0.8163, WT vs 115: *p* = 0.4753, WT vs 154 *p* = 0.0619, WT vs 622 *p* = 0.0715, F-value: 8.671, DF = 14, Matrin 3 *p-*values: WT vs 85: *p* = 0.9957, WT vs 115: *p* = 0.9722, WT vs 154 *p* = 0.9998, WT vs 622 *p* = 0.7550, F-value: 2.716, DF = 17). Full length blots are presented in Supplementary Figure 7. (**d**) Flow chart of IP-MS sample preparation and analysis protocols.

Previously, exome sequencing was used to identify four mutations in the RNA-binding protein Matrin 3 attributed to familial ALS: S85C, F115C, P154S and T622A^10^. Subsequently, five other groups discovered additional mutations in Matrin 3 linked to ALS^11–15^. The *MATR3* mutations predominately cluster in two potential hotspots found within amino acids 66–154 (containing six known mutations), and amino acids 610–787 (containing five known mutations) (Fig. 1a). S85C mutations in Matrin 3 have also been linked to vocal cord and pharyngeal weakness with distal myopathy (VCPDM), a progressive autosomal dominant distal myopathy that also results in dysphagia, dysphonia and vocal cord and pharyngeal weakness^16–18^. In human spinal cord tissue, Matrin 3 is predominantly localized within the nucleus of motor neurons; though in sporadic ALS (sALS) patients as well as a patient harboring the F115C Matrin 3 mutation, nuclear immunostaining was increased compared to non-neurologic disease controls, with occasional cytoplasmic immunostaining^10^. Rare Matrin 3 positive cytoplasmic inclusions have also been identified in patients harboring the C9orf72 repeat expansion as well as mutations in FUS^10,19^. Interactions between Matrin 3 and TDP-43 were also reported and this interaction was increased by the S85C mutation^10^.

Matrin 3 is an RNA-binding protein and a component of the nuclear matrix, and has been shown to be involved in diverse processes including the response to DNA damage^20^, mRNA stability^21^, RNA splicing^22^ and is phosphorylated in response to N-methyl-D-aspartate receptor (NMDAR) activation^23^ and murine Matrin 3 protein levels have been shown to be lowest in muscle and the spinal cord^24^. Recently, others have shown that expression of ALS linked mutations in Matrin 3 in a cell culture model does not result in gross mislocalization of the protein^25^. Due to the diverse roles of Matrin 3, we sought to identify functional alterations caused by ALS-linked mutations. Immunoprecipitation (IP) followed by tandem mass spectrometry (MS) experiments were performed to determine Matrin 3 protein-protein interactions (PPI) and any changes induced by disease-associated mutations. Using NSC-34 cells stably expressing either wild-type or mutant Matrin 3, we performed IP-MS and identified approximately 50 Matrin 3 interacting proteins with either wild-type or each mutant Matrin 3 protein. Multiple proteins within the TRanscription and EXport (TREX) protein complex that regulates mRNA nuclear export were found to interact with Matrin 3, and mutant Matrin 3 exhibited altered interactions with specific TREX proteins. We further demonstrate altered global mRNA nuclear export in cells expressing mutant Matrin 3 protein. Our results identify proteins that interact with wildtype and each mutant Matrin 3 protein, as well as a novel function of Matrin 3 in regulating mRNA nuclear export. These findings support a critical role for RNA processing and transport in the pathogenesis of ALS.

## Results

### Matrin 3 Protein-Protein Interactions (PPI) altered by ALS-Linked Mutations

While Matrin 3 performs many functions in the nucleus, few studies have identified proteins that interact with Matrin 3 and regulate its function^21,26^. To further define the functional role of Matrin 3 and how *MATR3* disease causing mutations alter its function, we examined protein-protein interactions of wild-type and each mutant Matrin 3 protein. Immunoprecipitation followed by tandem mass spectrometry experiments were performed using NSC-34 cells stably expressing either flag-tagged human wild-type or ALS associated mutant Matrin 3 proteins (Fig. 1b,c). While additional mutations in *MATR3* have recently been published, we chose to focus efforts on the original four mutations including a (chr5:138643358, C > G) resulting in a Ser85Cys (S85C) amino acid alteration, (chr5:138643448, T > G) Phe115Cys (F115C), (chr5:138643564, C > T) Pro154Ser (P154S), and (chr5:138658372, A > G) Thr622Ala (T622A) (Fig. 1a)^10^. A recent study demonstrated that mutant Matrin 3 protein remains predominately in the nucleus when transiently overexpressed in multiple cell types^25^. In our stable cell lines, these MATR3 mutations do not alter the cellular localization of endogenous or mutant Matrin 3, which remains predominantly nuclear (Fig. 1b). All four mutants were expressed at similar levels to wild-type and stable lines were selected to have low overexpression levels (approximately 2.5 fold) to stay close to physiological ranges. It was also noted that overexpression of mutant Matrin 3 did not lead to downregulation of the endogenous protein (Fig. 1c). Therefore, we examined Matrin 3 PPI specifically in the nucleus of cells expressing wild-type or mutant Matrin 3.

As outlined in Fig. 1d, nuclear extracts were prepared from each stable cell line and used for immunoprecipitation of exogenous Flag-tagged Matrin 3, followed by elution of bound proteins and identification by mass spectrometry (Supplemental Fig. 1). After peptide identification, interactions were analyzed by two methods, manual analysis and a probabilistic protein-protein interaction algorithm (SAINTexpress). Manual analysis consisted of filtering proteins to only include those that were identified in two out of three replicates at a fold change of 2.5 or greater over the maximum spectral counts identified in the empty vector control. SAINTexpress analysis was performed on proteins that were identified in two out of three replicates and at a fold change of 2.5 or greater as compared to the average spectral counts of empty vector, and yielded two populations of proteins; a medium confidence list of proteins that either met the manual analysis criteria or the SAINTexpress criteria of (AvgP > 0) and a high confidence list (AvgP > 0.7) (Fig. 1e). Overall, we identified approximately 300 proteins for wild-type and each mutant (range of 276–333), approximately 70 proteins that met the thresholding criteria for medium confidence (range of 61–87), and approximately 18 proteins that met the stringent criteria of high confidence interactors (range of 13–31). Across wild-type and all four mutants, a total of 167 unique proteins met the medium confidence threshold (Supplemental Table 1) and 53 proteins met the threshold for high confidence in at least one of the five cell lines (Table 1).

**Table 1:**
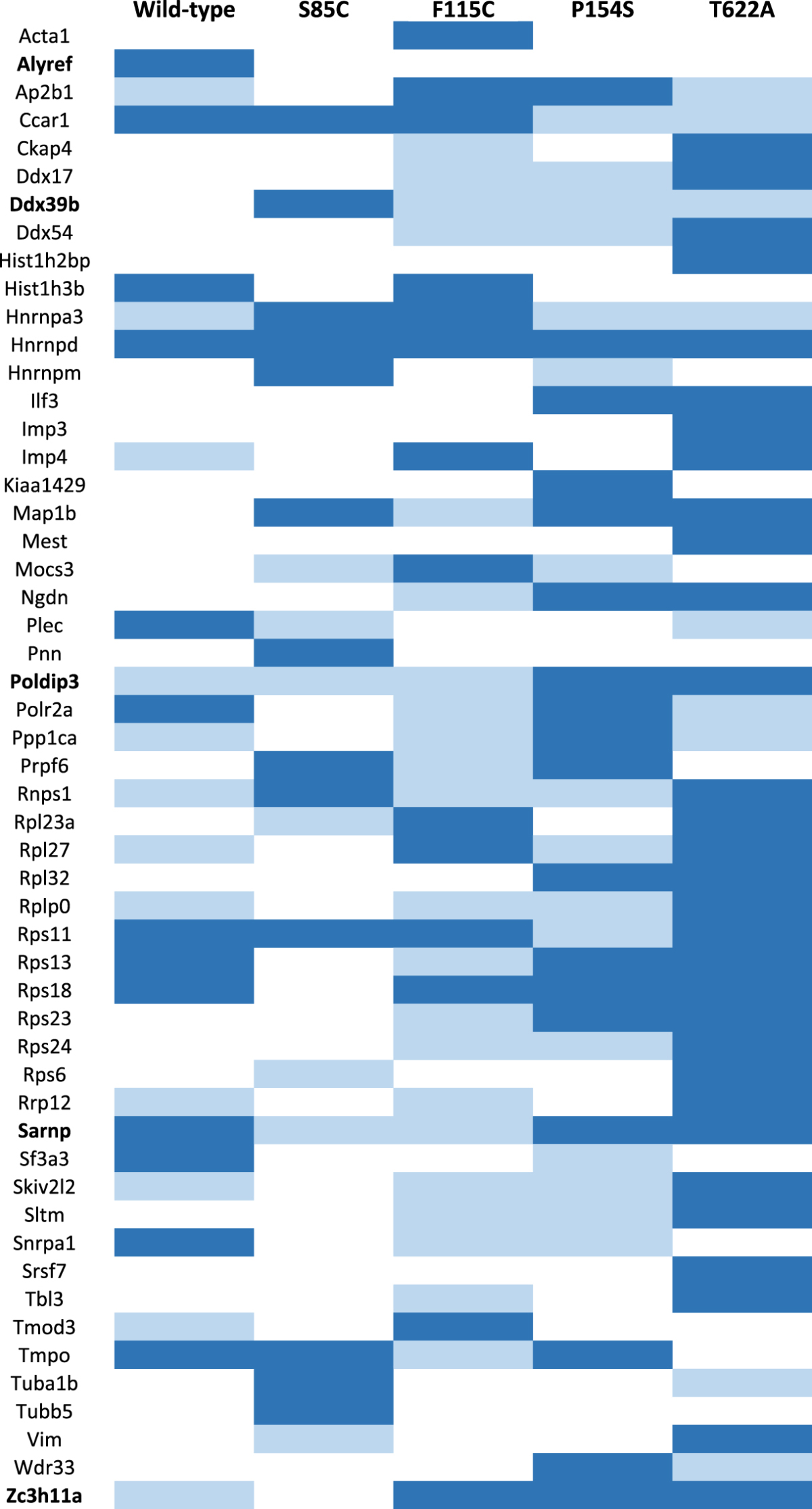
List of high confidence proteins identified by IP-MS. List of all proteins that met the threshold criteria for high confidence (identified in at least 2 replicates, fold change of at least 2.5 fold over vector and SaintExpress AvgP > 0.7) in wild-type or mutant Matrin 3 IP-MS experiments. Proteins shown in white for a particular cell line were either not identified or did not meet minimum threshold requirements for medium confidence (identified in at least 2 replicates, fold change of at least 2.5 fold over vector and SaintExpress AvgP > 0), proteins shown in light blue met medium confidence thresholds, proteins shown in dark blue met high confidence threshold. Protein names that are bolded denote proteins involved in nuclear export and/or the TREX complex.

### Gene Ontology Analysis Highlights mRNA Transport

ToppFun gene enrichment analysis was performed to functionally annotate the Matrin 3 interactome yielding a list of biological processes, many of which were RNA related (Table 2). The top biological process shared by wild-type and all four mutants was either “RNA processing” or in the case of Ser85Cys, “mRNA metabolic process”. For wild-type Matrin 3 PPIs the top 15 biological processes were all related to RNA, including mRNA and rRNA, processing and biogenesis, transcription and splicing. While the top gene ontology (GO) terms for all four Matrin 3 mutations included these processes, they also included terms involved in mRNA and RNA transport/localization, suggesting a role for Matrin 3 in RNA transport (Table 2).

**Table 2:**
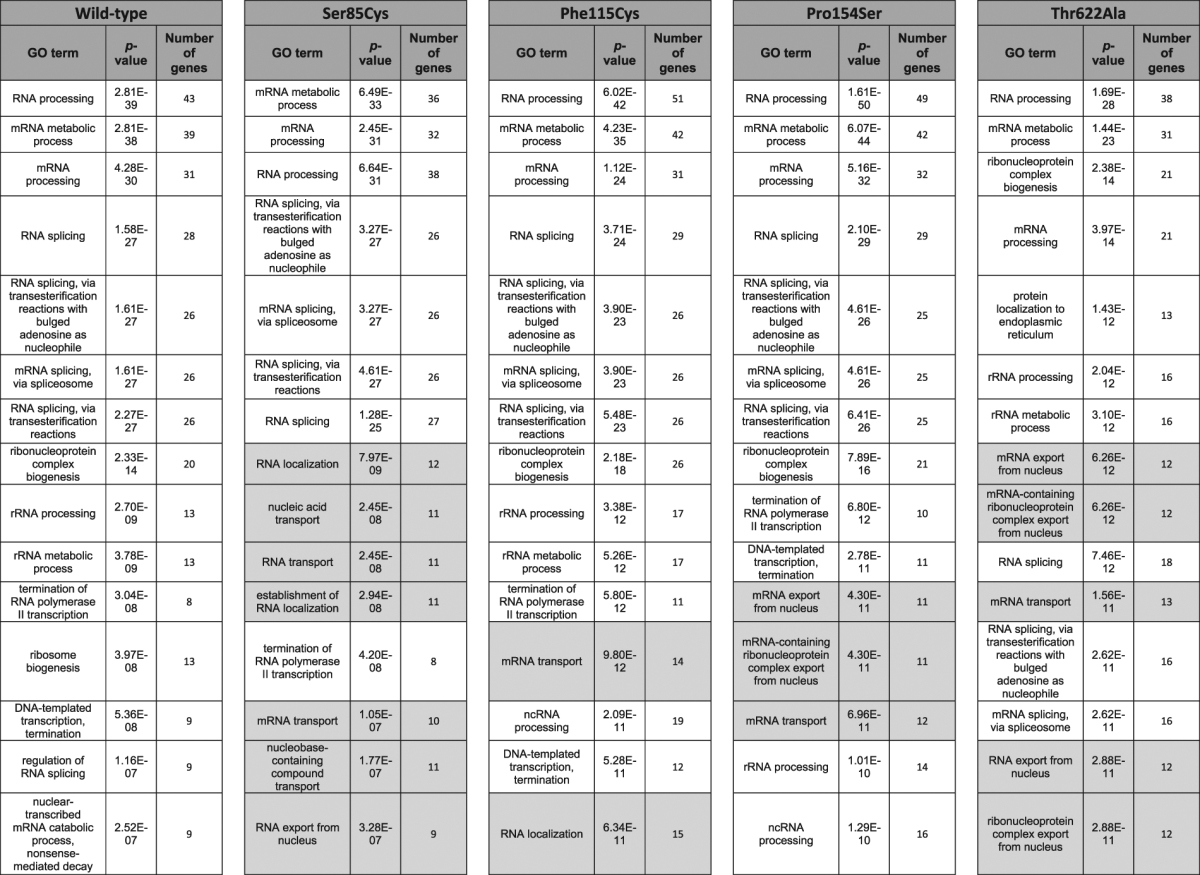
Gene ontology analysis for biological processes using medium confidence proteins identified by IP-MS. Top 15 biological processes by Bonferroni corrected *p*-value for wild-type and each mutant are listed. Grey boxes highlight terms involved in RNA transport and localization. Number of genes refers to the total number of Matrin 3 interacting proteins identified for each gene ontology term.

This pathway is particularly interesting due to recent reports describing interactions between the C9orf72 repeat and proteins involved in nuclear transport, as well as the subsequent defect in both protein import and RNA export in cells expressing the *C9orf72* repeat expansion^26–28^. Upon closer examination of Matrin 3 interacting proteins we identified multiple components and interactors of the TREX complex that controls mRNA nuclear export, including Aly (AlyRef), Sarnp (Cip29), Zc3h11a, Poldip3 and DdX39b (UAP56)^27,28^ (Table 1). We further explored and validated interactions of Matrin 3 with TREX proteins and the role of Matrin 3 in mRNA nuclear export.

**Figure 2:**
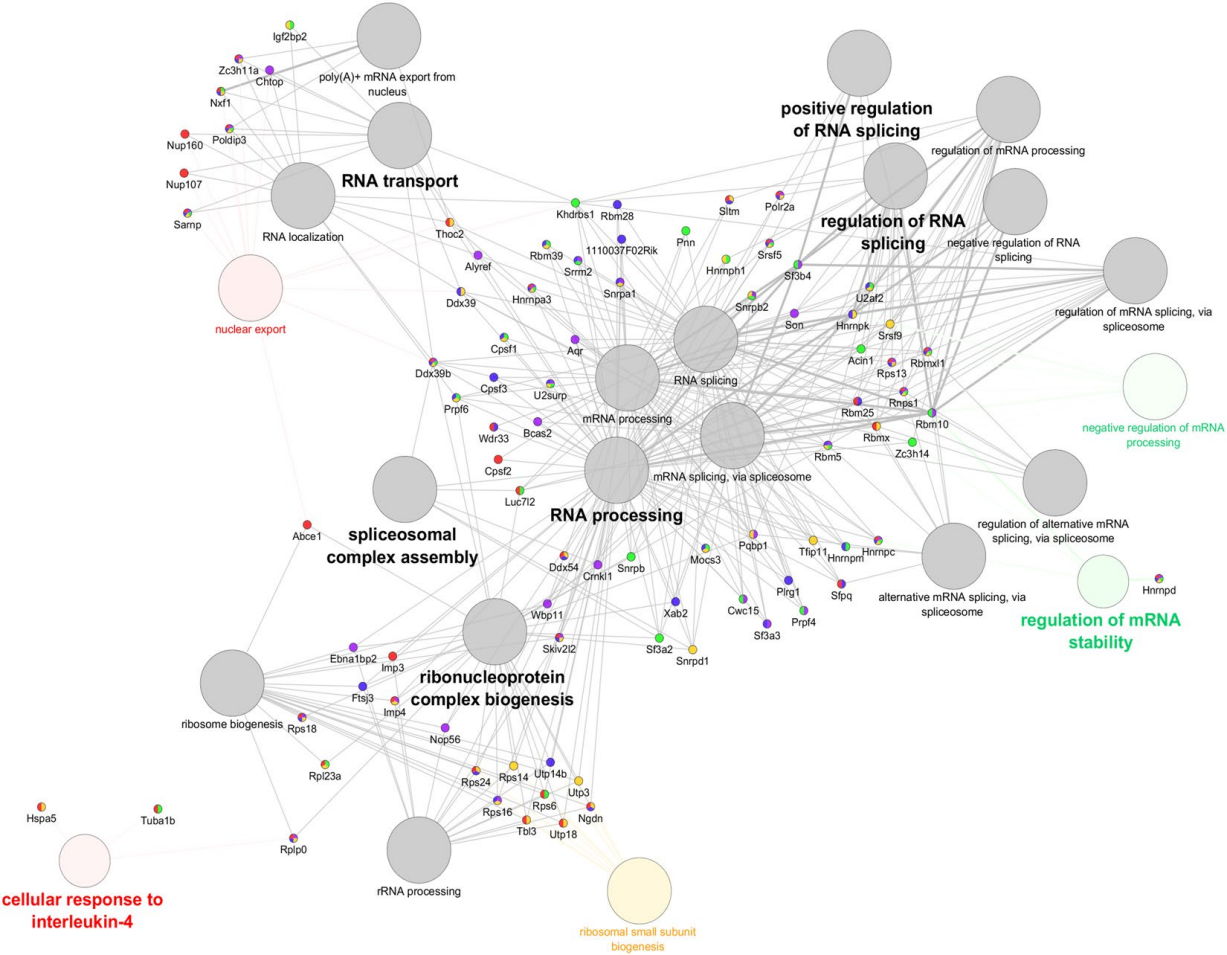
Functionally organized GO term network (ClueGO) of binding partners to wild type and mutant Matrin 3 in NSC-34 cells. Associated gene clusters and functional differences are highlighted. GO terms with a single sample frequency above 50% were color-coded: wild type (purple), Ser85Cys(green), Phe115Cys(yellow), Pro154Ser(blue), Thr622Ala(red), and unspecific (grey). Terms were considered unspecific if sample frequency was above 50% across more than one sample. Sample frequency was determined as a percentage based on the number of genes that defined that specific term. Increased size of GO term nodes inversely correlates to *p*-values computed by a two-sided hypergeometric test, with step-down Bonferroni correction.

ClueGO functional enrichment analysis was performed to aid in the visualization and interpretation of Matrin 3 PPI by grouping interacting proteins by biological processes, highlighting the role of Matrin 3 interacting proteins in RNA processing, RNA splicing, and RNP biogenesis (Fig. 2). This analysis also emphasizes the role of Matrin 3 in RNA transport with such GO terms as “poly (A) + mRNA export from the nucleus” and “RNA localization” common across all mutants and wild-type Matrin 3. While most GO terms were not unique to a specific mutation, “negative regulation of mRNA processing,” and “regulation of mRNA stability” were linked specifically to Ser85Cys; “cellular response to interleukin-4” and “nuclear export” were linked specifically to Thr622Ala; and “ribosomal small subunit biogenesis” was linked to Phe115Cys (Fig. 2). Future studies will explore the role of specific Matrin 3 mutations in mRNA stability and ribosomal biogenesis.

### Validation of Matrin 3 interactions with TREX proteins

**Figure 3:**
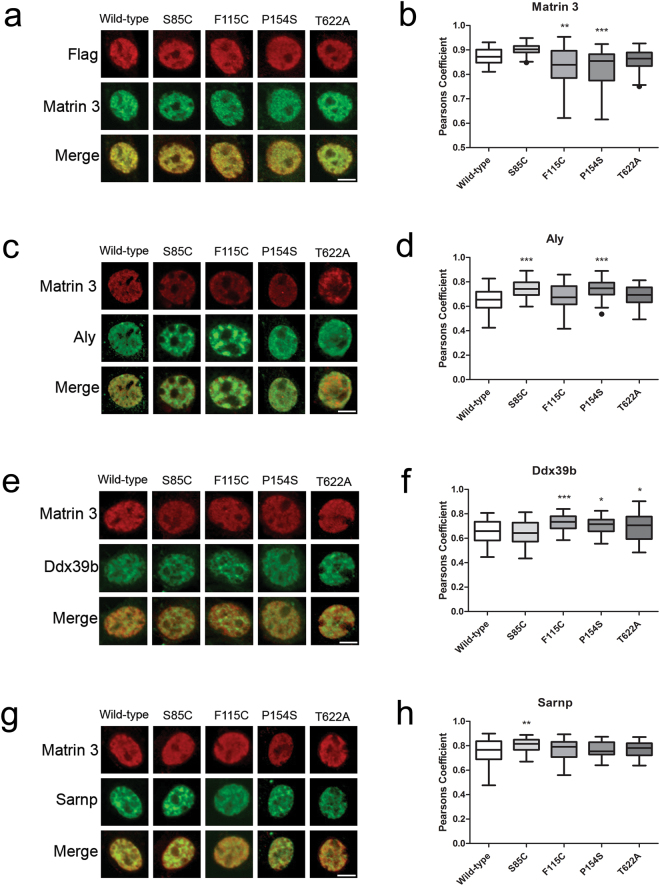
Immunofluorescence images of NSC-34 cells transiently transfected with wild-type or mutant Matrin 3 subjected to co-localization analysis. (**a**,**c**,**e**,**g**) Representative images from immunofluorescence staining are shown. In each case Flag is shown in red marking exogenous Matrin 3 and the protein of interest (Matrin 3, Aly, Ddx39b and Sarnp, respectively) is shown in green, merged image of two signals below. Scale bar indicates 5 µm. (**b**,**d**,**f**,**h**) Average Pearson’s correlation coefficient for Flag and the protein of interest, whiskers indicate 1.5 times the interquartile range (IQR) for 40–50 cells per genotype. One way ANOVA followed by Dunnett’s post-test, (*) denotes *p*-value < 0.05, (**) *p* < 0.01 and (***) *p* < 0.001 compared to wild-type (Matrin 3 *p-*values: WT vs 85: *p* = 0.1524, WT vs 115: *p* = 0.0035, WT vs 154 *p* = 0.0002, WT vs 622 *p* = 0.5535, F-value: 12.61, DF = 205; Aly *p-*values: WT vs 85: *p* = 0.0001, WT vs 115: *p* = 0.8307, WT vs 154 *p* = 0.0001, WT vs 622 *p* = 0.3368, F-value: 9.284, DF = 211; Ddx39b *p-*values: WT vs 85: *p* = 0.9725, WT vs 115: *p* = 0.0002, WT vs 154 *p* = 0.0107, WT vs 622 *p* = 0.0364, F-value: 7.701, DF = 224; Sarnp *p-*values: WT vs 85: *p* = 0.0090, WT vs 115: *p* = 0.9400, WT vs 154 *p* = 0.9791, WT vs 622 *p* = 0.8224, F-value: 2.913, DF = 228).

IP-MS results were validated in two manners, co-immunoprecipitation followed by western blot (IP-WB) and double-label immunofluorescence microscopy of cultured cells. Both methods also provided relative quantification of protein interactions with wild-type or mutant Matrin 3. Immunofluorescence microscopy was performed on cells transiently transfected with either wild-type or mutant Matrin 3 and immunostained with antibodies against the Flag tag on Matrin 3 and the protein of interest. Pearson’s correlation coefficients were calculated for nuclear immunostaining to quantify levels of co-localization. Co-immunofluorescence against both Flag and Matrin 3 allowed us to explore whether mutant Matrin 3 co-localizes with endogenous Matrin 3 throughout the nucleus. Pearson’s correlation coefficients ranged from 0.8 and 0.9 for endogenous Matrin 3 compared to exogenous Flag-Matrin 3 indicating high levels of co-localization between the two. The F115C and P154S mutations of Matrin 3 co-localized significantly less with endogenous Matrin 3, suggesting that ALS-linked mutations alter the localization of mutant protein within the nucleus (Fig. 3a,b, Supplemental Fig. 3). This change in mutant Matrin 3 distribution within the nucleus may reflect the altered protein-protein interactions observed by IP-MS and contribute to disease pathogenesis induced by these mutations.

We next explored co-localization between proteins involved in TREX regulated mRNA export (Aly, Sarnp and Ddx39b) with wild-type and mutant Matrin 3. For all three proteins, Pearson’s coefficients were found to be 0.6 or higher, implying that the three proteins do co-localize with Matrin 3, further validating our IP-MS results (Fig. 3c–h). The S85C and P154S mutations in Matrin 3 exhibited increased levels of co-localization with Aly as compared to wild-type Matrin 3 while F115C and T622A levels remained similar to wild-type (Fig. 3c,d, Supplemental Fig. 4). Three of the four Matrin 3 mutations (F115C, P154S, and T622A) exhibited increased co-localization between mutant Matrin 3 and Ddx39b, with S85C showing similar levels to wild-type (Fig. 3e,f, Supplemental Fig. 5). S85C did, however, show increased levels of co-localization with Sarnp while the other mutants showed no difference (Fig. 3g, h Supplemental Fig. 6). Taken together, ALS-linked mutations in Matrin 3 co-localized less with endogenous Matrin 3 and exhibited increased co-localization with components of the TREX complex. A shift in protein interactions may induce alterations in TREX function within cells expressing ALS associated mutations in Matrin 3.

**Figure 4:**
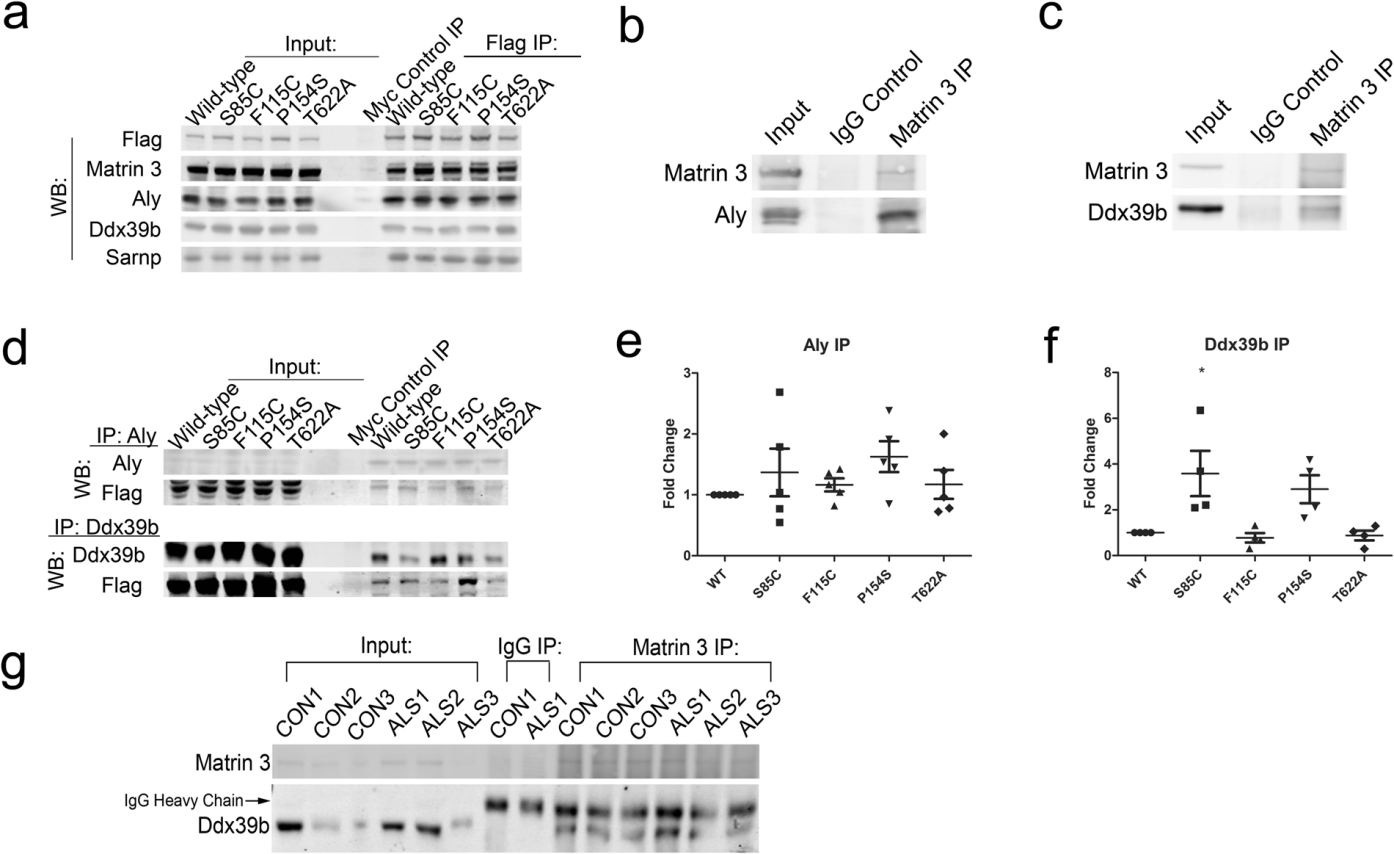
Immunoprecipitation followed by western blot from NSC-34 cell lines and human lumbar spinal cord tissue. (**a**) Immunoprecipitation using Flag antibody followed by western blot which was probed with Flag and Matrin 3 to confirm efficient pull down of Matrin 3, or TREX components Aly, Ddx39b and Sarnp; representative blots are shown, and all experiments were performed a minimum of three times with similar results. (**b**,**c**) Matrin 3 IP performed on endogenous Matrin 3 in untransfected NSC-34 cells. Immunoblots are probed with Aly (**b**) or Ddx39b (**c**). (**d**) Reverse immunoprecipitation experiments using antibodies against Aly and Ddx39b followed by western blot probed with either Aly or Ddx39b confirming pull-down of the target and Flag to measure the amount of mutant Matrin 3 bound, representative blots shown. (**e**,**f**) Quantification of Aly and Ddx39b IP-WB experiments; values are expressed as Flag signal over signal of the bait protein (Aly or Ddx39b respectively) to control for IP efficiency, Aly IP values from five replicates, Ddx39b IP values from four replicates. Values are expressed as fold change over wild-type and error bars represent SEM. One way ANOVA followed by Dunnett’s post-test, (*) denotes *p*-value < 0.05 (Aly *p-*values: WT vs 85: *p* = 0.6561, WT vs 115: *p* = 0.9670, WT vs 154 *p* = 0.2250, WT vs 622 *p* = 0.9611, F-value: 1.011, DF = 24; Ddx39b *p-*values: WT vs 85: *p* = 0.0133, WT vs 115: *p* = 0.9944, WT vs 154 *p* = 0.0760, WT vs 622 *p* = 0.9993, F-value: 6.025, DF = 19).(**g**) Matrin 3 IP performed in human lumbar spinal cord nuclear lysates of controls n = 3 and ALS patients n = 3. Immunoblot is probed with Ddx39b and Matrin 3. Arrow indicates IgG heavy chain band. Patient demographics can be found in Supplemental Table 2. Full length blots presented in Supplementary Figure 7.

Using co-IP coupled with western blot, Matrin 3 interactions were confirmed for each of the TREX proteins (Fig. 4a). We also confirmed protein-protein interactions between endogenous Matrin 3 and Aly and Ddx39b (Fig. 4b,c). To rule out potential non-specific pull-down by the Flag antibody, reverse IP experiments were performed using antibodies against Aly or Ddx39b. Our results confirm the predicted interaction of Matrin 3 with Aly and Ddx39b (Fig. 4d). Quantitation of these blots showed a trend towards increased binding of both Aly and Ddx39b to the S85C and P154S mutations in Matrin 3, while increased binding of S85C Matrin 3 to Ddx39b reached statistical significance (Fig. 4e,f).Co-immunopreciptiation between Matrin 3 and Ddx39b was also performed in human post-mortem lumbar spinal cord tissue, confirming that these proteins interact *in vivo* and in the context of sporadic ALS (Fig. 4g).

### Matrin 3 mutations reduce mRNA nuclear export

**Figure 5:**
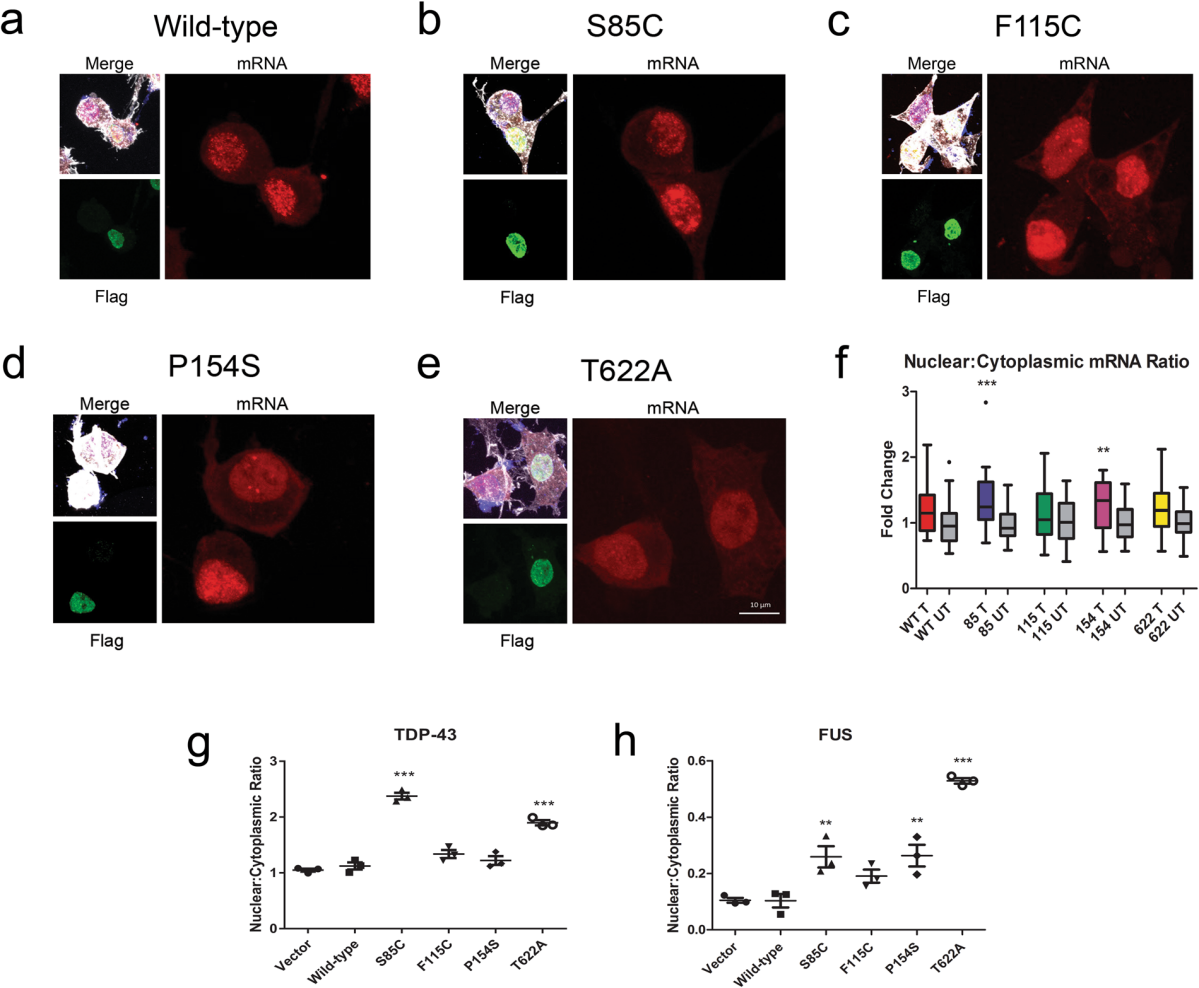
RNA-FISH and cellular fractionation followed by RT-PCR show defects in RNA export. Experiments performed in NSC-34 cells transiently transfected with either wild-type or mutant Matrin 3. (**a**–**e**) mRNA signal of RNA-FISH experiment shown in red, immunofluorescence staining of cells using actin to mark the cell body (white), Flag to mark transfected cells (green) and DAPI to mark the nucleus (blue) (representative images). (**f**) Nuclear to cytoplasmic mRNA ratio of transfected (T) vs. untransfected (UT) cells for wild-type Matrin 3 and each mutant expressed as fold change over untransfected cells on the same slide, 31–34 cells were measured per genotype collected from three independent experiments. Whiskers indicate 1.5(IQR). One way ANOVA followed by Bonferroni post-test, WT *p-*value: 0.0697, 85 *p-*value: 0.0005, 115 *p-*value: 0.4100, 154 *p-*value: 0.0073, 622 *p-*value: 0.0596). (**g**,**h**) Cell fractionation followed by RT-PCR on nuclear and cytoplasmic fractions of HEK-293 cells. Values are expressed as average nuclear to cytoplasmic ratio of either TDP-43 or FUS mRNA, normalized to tRNA-Lys for the nuclear fraction and cytochrome b for the cytoplasmic fraction. Error bars represent mean and SEM of three replicates. Experiments were each performed three times, graphs show representative experiment. One way ANOVA followed by Dunnett’s post-test (TDP-43 *p-*values: WT vs Vector: *p* = 0.8529, WT vs 85: *p* = 0.0001, WT vs 115: *p* = 0.1003, WT vs 154 *p* = 0.6840, WT vs 622 *p* = 0.0001, F-value: 74.31, DF = 17; FUS *p-*values: WT vs Vector *p* = 0.9999, WT vs 85: *p* = 0.0054, WT vs 115: *p* = 0.1336, WT vs 154 *p* = 0.0045, WT vs 622 *p* = 0.0001, F-value: 35.21, DF = 17, (*) denotes p-value < 0.05, (**) p < 0.01 and (***) p < 0.001 for both RNA FISH and RT-PCR data.

Unlike other nuclear export machinery, the TREX complex is restricted to the export of mRNA^29^. To confirm the role of Matrin 3 in nuclear mRNA export we performed fluorescence *in situ* hybridization (mRNA-FISH) using an oligo dT probe against poly(A) containing mRNA in cells transiently expressing either wild-type or mutant Matrin 3, and measured the amount of mRNA in the nucleus and cytoplasm of each cell. Nuclear to cytoplasmic ratios of mRNA were calculated for cells expressing mutant Matrin 3 (Transfected), and neighboring non-transfected cells (Untransfected) (Fig. 5a–f). There was a trend towards an increased nuclear to cytoplasmic ratio of mRNA for transfected vs. non-transfected for wild-type and all mutant expressing cells. There was a statistically significant increase in polyA-mRNA nuclear to cytoplasmic ratio in cells expressing S85C and P154S Matrin 3 (Fig. 5f). Expression of S85C resulted in a 34% increase in the nuclear to cytoplasmic ratio of mRNA and P154S expression resulted in a 29% increase in the ratio (Fig. 5f). The increased nuclear to cytoplasmic ratio implies that mRNA is sequestered within the nucleus in cells expressing mutant Matrin 3 protein and S85C and P154S Matrin 3 mutations induce significant defects in mRNA nuclear export.

### Mutations in Matrin 3 lead to export defects of TDP-43 and FUS mRNA

After demonstrating a global defect in mRNA export from the nucleus, we explored whether this defect affected specific mRNAs for proteins relevant to ALS. We focused on the mRNA of TDP-43^10^ and FUS^30^, two RNA-binding proteins previously linked to ALS, both of which have been shown to bind to Matrin 3. To demonstrate the role of Matrin 3 in the nuclear export of TDP-43 and FUS mRNA, cellular fractionation followed by RT-PCR for TDP-43 and FUS mRNA in each compartment was performed using HEK-293 cells expressing either wild-type or ALS associated mutant Matrin 3. HEK-293 cells were utilized in this experiment to demonstrate the role of Matrin 3 in nuclear export in a human cell line, though similar results were obtained using the NSC-34 cell lines (data not shown). Expression of wild-type Matrin 3 generated no change when compared to control cells but expression of S85C, and T622A Matrin 3 mutations led to an increase in the nuclear to cytoplasmic ratio of TDP-43 mRNA compared to an empty vector control (Fig. 5g). The nuclear to cytoplasmic ratio of FUS mRNA was increased by expression of S85C, P154S and T622A mutant Matrin 3 (Fig. 5h). In both cases RT-PCR with primers specific for TDP-43 and FUS was also performed on whole cells to confirm that the difference in mRNA levels was not due to differential mRNA expression or degradation (Supplemental Fig. 2).

## Discussion

We performed IP-MS to identify Matrin 3 interacting proteins, and determine alterations in PPIs caused by ALS-linked mutations. This yielded 167 total proteins that met our confidence threshold for interacting with either wild-type or one of the four mutant Matrin 3 proteins (Supplemental Table 1). This contrasts with previously published IP-MS experiments which each identified only eight wild-type Matrin 3 interacting proteins^21,26^. Our results identified 6 of the 8 proteins identified in Salton *et al*., and 7 out of the 8 proteins identified in Erazo *et al*., though these proteins did not meet our stringent confidence thresholds for protein interaction. In comparison to other studies, the discrepancies in the number of Matrin 3 interactors identified in our work is likely due to the different cell types and methodologies used for the proteomic analysis. We also identified differences in PPIs induced by disease causing mutations in *MATR3*. On average, each mutant Matrin 3 protein exhibited approximately 60% different interactors than the wild-type interactome (ranging between 54–63%), suggesting that these mutations allow for novel Matrin 3 protein interactions that may impact its function and contribute to disease.

Gene ontology analysis highlighted terms including “RNA localization,” “RNA transport,” and “mRNA transport” within the top 15 most enriched biological processes for mutant but not wild-type Matrin 3. Examination of Matrin 3 PPIs involved in RNA localization, transport and export yielded several members of the TREX complex. The role of the TREX complex is to link transcription, mRNA processing and mRNA nuclear export. After transcription, pre-mRNA molecules associate with several proteins to form a dynamic messenger ribonucleoprotein (mRNP). TREX proteins are components of this mRNP from the initial stages of transcription, throughout splicing and processing, and ultimately to delivery of the mRNP for transfer to the nuclear pore and export^31^. Interestingly, we identified Matrin 3 interactions not only with proteins that are core components of TREX such as Aly, Sarnp, Ddx39b, Zc3h11a, Chtop, Poldip3, and Thoc2, but also interactions between Matrin 3 and proteins involved in all stages of mRNA biogenesis and export (Supplemental Table 1). Matrin 3 also interacted with three RNA polymerase II subunits, Polr2a, Polr2b, and Polr2c that function at early steps of transcription. Proteins involved in RNA splicing that interact with Matrin 3 include Pnn^32^, Prpf6^33^, Sf3a3^34^, Skiv2l2^35^, Snrpa1^36^, and Srsf7^37^. Interestingly Pnn, Prpf6 and Srsf7 bind to various mutations in Matrin 3 but were not found on the wild-type list. While we have not explored the role of Matrin 3 in mRNA splicing, others have shown a role for wild-type Matrin 3 as a splicing regulator which tends to repress exon inclusion^22^. One of the transcripts shown to be regulated by Matrin 3 was ADAR1B which is altered in ALS^38^. Future work will explore the role of wild-type and mutant Matrin 3 in RNA splicing.

We also identified Matrin 3 interactions with proteins involved in the delivery of mRNPs from TREX to the nuclear pore complex, including interactions with the nuclear export receptor NXF1, and nuclear pore proteins Nup107 and Nup160. While we have not validated these mass spectrometry based results, our data suggests that Matrin 3 participates in processes throughout mRNA processing, transport and export from the nucleus. It is unclear whether Matrin 3 performs these functions as a resident nuclear matrix protein or if there is a soluble pool of Matrin 3 that functions within and travels with TREX. We determined that mutant Matrin 3 proteins are less co-localized with endogenous Matrin 3 (when compared to exogenously expressed wild-type Matrin 3) and instead more co-localized with TREX proteins Aly, Ddx39b and Sarnp. This suggests that while there are no gross overall changes in the localization of mutant Matrin 3 within the cell, there is a re-distribution in the localization of mutant Matrin 3 within the nucleus. Altered protein-protein interactions with TREX proteins may explain the observed defects in mRNA nuclear export induced by Matrin 3 mutant proteins in our study. The two Matrin 3 mutations with the strongest global export defects, S85C and P154S, were also the two mutations that showed increases in co-localization with Aly and increased binding to both Aly and Ddx39b. This suggests that alterations in the associations of Aly and Ddx39b with Matrin 3 caused by ALS-linked mutations may be key to the downstream phenotype of nuclear mRNA retention. Future studies will define the role of Matrin 3 interactions with Aly and Ddx39b in regulating mRNA nuclear export. In addition, prior studies have shown a role for Matrin 3 in the nuclear export of HIV transcripts via CRM1 mediated nuclear export^39^. Since CRM1 mediated nuclear export can also export some mRNAs, future studies will also explore the potential roles of Matrin 3 mutations in modulating RNA nuclear export of retroviral infected cells. We previously reported increased binding between the S85C Matrin 3 mutant protein and TDP-43, suggesting this mutation could have increased affinity to many different nuclear proteins^10^. Interestingly, the S85C Matrin 3 also showed the largest change in the nuclear to cytoplasmic ratio of TDP-43 mRNA (Fig. 5g), and patients with this genotype have been shown to exhibit ALS and distal myopathy phenotypes^10,16,17,40^. While various Matrin 3 mutations impact TDP-43 or FUS mRNA nuclear export to various degrees, we did not detect global changes in TDP-43 or FUS protein levels or subcellular distribution in these same cells (data not shown).

The cell culture model utilized in these experiments (NSC-34) is a mouse motor neuron like hybrid cell line. Matrin 3 is highly conserved between human and mouse with 98.5% sequence homology at the amino acid level and 94.8% at the DNA level (HomoloGene, NCBI). Three of the four mutations that were studied in this work are in highly conserved regions of Matrin 3 (S85C, P115C, P154S), however the sequence differs at amino acid 622, which is threonine in humans and alanine in mice. While this is a limitation of the model system used in this study, IP-MS experiments were also performed in a human cell line (HEK-293) and identical interactions between Matrin 3 and TREX proteins including Chtop, Aly, and Zc3h11a were observed in this human cell line (data not shown). We also demonstrate interactions of endogenous Matrin 3 with TREX proteins and interactions of wildtype Matrin 3 with the TREX protein Ddx39b in human post-mortem tissue samples (Fig. 4). Future studies will further explore interactions of mutant Matrin 3 and TREX proteins in human post-mortem tissue samples or patient derived stem cells.

While a role for Matrin 3 in TREX and nuclear export is novel, a functional role for Matrin 3 in splicing has been recently demonstrated^22^, and splicing is an integral functional role of TREX. Matrin 3 was linked to the export of viral RNA via the CRM1 mediated nuclear export pathway^41^, as discussed above. Finally, Matrin 3 was identified by mass spectrometry using isolated nuclear pore fractions^42^, suggesting that this protein can be located at the nuclear pore. The role of nuclear transport in ALS was initially implicated due to the discovery of mutations in the export protein GLE1 in familial ALS^43^. More recently, nucleocytoplasmic transport has moved to the forefront of ALS pathobiology due to nuclear transport defects in numerous model systems expressing either the *C9orf72* repeat expansion or dipeptide repeat proteins (DPRs)^44–46^ as well as in cells expressing c-terminal fragments of TDP-43^47^. Proteins that modified the C9orf72 phenotype were found to be interactors of Matrin 3 in our study including Aly, Nup 107, and Nup 160^44^. Though most studies have suggested a deficiency in the import of proteins, a similar nuclear accumulation of mRNA was seen in cells expressing the G_4_C_2_ repeat^44^, as well as cells expressing TDP-43 c-terminal fragments^47^, suggesting a defect in nuclear export of RNA. While the mechanism by which either the C9orf72 repeat expansion, TDP-43 c-terminal fragments and *MATR3* mutations result in mRNA export defects is unknown, the fact that Matrin 3 interacts with both suggests that either all three mutations impact the same functional pathway, or this defect is mediated by interactions with one another^10,48,49^. This link between Matrin 3 and C9orf72 is further supported by the finding of rare Matrin 3 positive inclusions in a patient with a C9orf72 repeat expansion but not in other sALS cases^10,19^.

In this study, we demonstrated binding of Matrin 3 to 167 total proteins with 53 that met high confidence thresholds for protein interactions, greatly increasing the known Matrin 3 PPIs. This is the first study describing the PPI of the ALS-associated Matrin 3 mutations S85C, F115C, P154S and T622A. Importantly, our results demonstrate a novel role for Matrin 3 in mRNA nuclear export, possibly mediated via direct interactions with proteins of the TREX complex. Disease causing mutations in *MATR3* alter interactions with TREX proteins and nuclear export of mRNA, further highlighting the role for mRNA processing and nuclear export in the pathogenesis of ALS.

## Methods

### Immunoprecipitation and Western Blot

Flag immunoprecipitations (IP) were performed using NSC-34 cells stably expressing 3x Flag Matrin 3 wild-type or ALS-associated mutant, endogenous IPs were performed in untransfected NSC-34 cells. Immunoprecipitations were performed on nuclear fractions (400 µg of total protein), isolated using a Nuclear Complex Co-IP Kit (Active Motif) with minor modifications, and either Flag M2 affinity gel (Sigma-Aldrich) or antibodies against Ddx39b, Aly or Matrin 3.

Lumbar spinal cord tissue homogenates were prepared from frozen tissue from controls (*n* = 3) and ALS cases (*n* = 3) for co-immunoprecipitation studies. Nuclear and postnuclear extracts were prepared as described previously^50^. Briefly, samples were homogenized in a solution containing 10 mM Tris (pH 8.0), 10 mM MgCl_2_, 15 mM NaCl, and 0.1% Ipegal CA-630 (Sigma) supplemented with protease and phosphatase inhibitors, and nuclei were collected via low-speed centrifugation at 800 × *g* for 5 min. Nuclei were lysed in buffer containing 0.42 M NaCl, 20 mM HEPES, 20% glycerol and 0.1% Ipegal CA-630 supplemented with protease and phosphatase inhibitors. Nuclear lysate was collected after a 10 min lysis by centrifugation at 14,000 rpm for 5 min. The resulting supernatant was saved as the nuclear extract and used for immunoprecipitation (150 μg protein per sample). After eluting proteins, the mixture was separated using gel electrophoresis on NuPage 4–12% Bis-Tris gels (Thermo Fisher) and either transferred to Immobilon FL polyvinylidene difluoride (PVDF) membranes (Millipore) or stained with Coomassie blue (BioRad) for mass spectrometry analysis. For IP followed by western blot (IP-WB) membranes were blocked in Odyssey blocking buffer (LiCor) and probed with the indicated primary antibody overnight followed by the appropriate secondary antibody (LiCor). Both WB and Coomassie stained membranes were imaged on an Odyssey CLx imager (LiCor).

### Mass Spectrometry

#### In-gel digestion

After IP followed by electrophoresis (see above) lanes were excised into individual bands, excluding heavy and light IgG chains observed at 52kDA and 25 kDa respectively. Bands were cut into 1–2mm^3^ cubes and processed using published methods^51^. Briefly, resulting fractions were reduced with 10 mM DTT (6 °C for 30 min), alkylated with 55 mM iodoacetamide (room temperature for 30 min, in the dark) and digested using 20 ng/mL of Trypsin Gold (Promega) (37 °C, overnight). Finally, peptides were extracted, vacuum dried and stored at −20 °C until LC-MS analysis.

#### LC-MS analysis

Individual fractions were reconstituted in 0.1% formic acid and analyzed using online liquid chromatography on a Waters nanoAcquity UPLC coupled to a Thermo LTQ Orbitrap Velos mass spectrometer. Chromatography solvents A and B were 0.1% formic acid in water or acetonitrile, respectively. Peptides were first trapped on a 30 mm × 100 μm diameter fused silica column packed with 3 µm 120 Å ReproSil-Pur C18 AQ resin (Dr Maisch GMBH, Ammerbuch-Entringen, Germany) at 7.5 μl/min and 5% solvent B, before separation at 500 nl/min on a 100 mm × 100 μm analytical column (same solid phase as trap column) in a 3–40% solvent B gradient for 17 min, followed by 40–90% in 0.6 min, then 90% B for 2 min and final re-equilibration for 10.5 min. The mass spectrometer was operated in positive ion mode using a spray voltage of 1.8 kV, and a capillary temperature of 200 °C. Data were acquired in top-15, data-dependent acquisition mode using a collision voltage of 30 V.

### Protein Identification

The raw mass spectra were deconvoluted using Proteome Discoverer v1.4.1.14 (Thermo Fisher Scientific, Waltham, MA). The spectra were searched against *Mus Musculus* (Swissprot, January 2015) supplemented with human Matrin-3 using Mascot v2.4.1 (Matrix Science, London, UK) with the following variable modifications: oxidation (Met) and carbamidomethyl (Cys). Mass tolerances for precursor ions were set at ± 10 ppm, for fragment ions at ± 0.8 Da. A maximum of 2 missed cleavages was allowed. Data were processed for label-free quantitation using Scaffold v4.5.1 (Proteome Software Inc., Portland, OR) and X!Tandem (The GPM, v2010.12.01.1) to further improve confidence in protein identification. At least 2 peptides were required for protein identification, with 0.1% peptide FDR and 1% protein FDR. Only exclusive spectral counts were used for prediction of protein-protein interactions.

### Bioinformatics and Pathway Analysis

Probabilistic scoring of protein-protein interaction (PPI) combined with manual thresholding analysis of interactants to wild-type and mutant forms of Matrin-3 was performed using SAINTexpress^52^. Using SAINTexpress, average probability (AvgP), fold change and a Bayesian False Discovery Rate (BFDR) were computed for each interaction pair. Fold-changes were calculated using average exclusive spectral counts against the empty vector as a background control for non-specific binding. PPIs were filtered by presence in at least 2 out of 3 replicates, fold change ≥2.5 and AvgP ≥ 0.7. For manual analysis, Matrin-3 interactors were filtered by presence in at least 2 out of 3 replicates and a fold change ≥2.5 based on maximum spectral counts.

#### Immunofluorescence and RNA FISH

Immunofluorescence staining was performed on NSC-34 cells either transiently or stably expressing Matrin 3 constructs. Cells were grown on glass coverslips, fixed in 4% paraformaldehyde (PFA) in PBS for 5 min, then permeabilized in 0.1% Triton X-100 for 5 min. Cells were then blocked for one hour in SuperBlock (Scytek), and primary antibody was added for either one hour at room temperature or overnight at 4 °C followed by the appropriate secondary antibody for one hour at room temperature. Nuclei were labeled with 4, 6-diamidino-2-phenylindole (DAPI) for 5 min (Invitrogen). For co-localization analysis, Pearson’s correlation coefficients were calculated using Imaris software (Bitplane) first by masking the DAPI channel to measure only co-localization within the nucleus, and then applying an automatic thresholding algorithm. The number of cells analyzed over the course of three independent experiments are as follows: Matrin 3-WT = 41, 85 = 43, 115 = 40, 154 = 41, 622 = 41, Aly-WT = 41, 85 = 40, 115 = 42, 154 = 46, 622 = 43, Ddx39b-WT = 50, 85 = 43, 115 = 45, 154 = 44, 622 = 43, Sarnp-WT = 46, 85 = 50, 115 = 44, 154 = 42, 622 = 47.

RNA FISH experiments were performed on NSC-34 cells transiently expressing Matrin 3 constructs. Cells were plated on glass coverslips, fixed in 4% PFA for 10 min followed by permeabilization in 0.2% Triton X-100 for 10 min and washes in 70% ethanol and 1 M Tris-HCl. Cells were hybridized in buffer containing 2 ng/µl Cy3 labeled Oligo dT, 0.5 µg/µl tRNA, 2 µg/µl BSA fraction V, 10% dextran sulphate, 20% formamide, and 2x saline-sodium citrate (SSC) buffer for 3 hours at 37 °C in a humidified chamber. Cells were then washed in SSC and subjected to the same immunocytochemistry protocol as listed above beginning with blocking step. The number of cells analyzed over the course of three independent experiments are as follows: WT transfected = 34, WT untransfected = 31, 85 transfected = 34, 85 untransfected = 33, 115 transfected = 32, 115 untransfected = 33, 154 transfected = 32, 154 untransfected = 32, 622 transfected = 33, 622 untransfected = 33.

Images were captured using a Zeiss LSM 710 confocal microscope and image analysis was performed using Imaris software (Bitplane).

#### Gene Ontology

Gene ontology assessments were performed using ToppGene Suite (ToppFun). Medium confidence lists of proteins were utilized (identified in at least 2 replicates with a fold change of at least 2.5 over controls, and AvgP > 0 using the SAINTexpress program) for this analysis. Calculations were made using GO: Biological Processes with a FDR p-value cutoff of 0.01. The top ten results for each group are shown in order along with p-values and the number of proteins identified in each biological process.

To create a visual network of overlapping and unique GO terms across the Matrin-3 mutant samples, ClueGO v2.3.2 was utilized through Cytoscape v3.3.0. The proteins were aligned to GO:Biological Processes using the mouse proteome. GO term fusion was implemented merging parent-child terms with shared proteins. All other default ClueGO parameters were used. Proteins shared between identified GO terms were selected for display on the network map^53^.

#### Cell Culture and Creation of Matrin 3 stable lines

NSC-34 cells (Cellutions Biosystems) were cultured in DMEM supplemented with 10% FBS and grown in the presence of 1% Pen-Strep at 37 °C and 5% CO_2_. Matrin 3 cDNA plasmid HsCD00075976 was obtained from the DNASU plasmid repository at Arizona State University. Matrin 3 3x Flag constructs were PCR amplified using Phusion High-Fidelity Polymerase (NEB) then sub-cloned into a pcDNA3 vector (Invitrogen) along with 3 Flag peptides attached to the N-terminus of the protein. Constructs expressing ALS linked mutations were created by performing site directed mutagenesis (Agilent Technologies) on Matrin 3 3x Flag pcDNA3 constructs^54^. Cells were transfected using Lipofectamine 3000 (Life Technologies) and stable lines were selected under the using 500 µg/ml Genetecin (Life Technologies) applied 24hrs after transfection. For transient transfections cells were used 48 hrs after transfection.

### Nuclear/Cytoplasmic RNA fractionation

HEK-293 cells were harvested and processed for nuclear and cytoplasmic RNA fractionation as described in^55^ adapted from the method developed by^56^. Briefly, cells were transfected with the various Matrin 3 constructs, and harvested 48h later. Pellets were rinsed in PBS and resupended in lysis buffer A (10 mM HEPES, 1.5 mM MgCl2, 10 mM KCl, 0.5 mM DTT and 2 mM vanadylriboside VRC). A fraction was immediately separated for the total RNA fraction, and the remaining fraction was incubated on ice, and broken down with a chilled Dounce homogenizer to release nuclei. Cells were then spun down at 228xg for 5 min to release the cytoplasmic fractions (supernatant) and the nuclei (pellets). Nuclei were washed in buffer A twice, resuspended in Buffer S1 (250 mM sucrose, 10 mM MgCl2 and 2 mM VRC), and layered on top of a cushion of buffer S3 (880 mM sucrose, 0.5mMMgCl2, 2 mM VRC). Nuclei were spun down at 2800xg and for 10 min and pellets were resuspended in buffer A. Trizol was then added to all fractions, and RNA was extracted using the Direct-zol RNA miniprep kit (Zymo Research, Irvine, CA). cDNA was synthesized using Superscript VILO (ThermoFisher Scientific), and cDNA was used for quantitative real-time PCR using PowerUp Sybr Green master mix. All curves were normalized by the comparative ΔΔCt method. Nuclear fraction RNA levels were normalized to tRNA-Lys (For: CGGATAGCTCAGTCGGTAGA and Rev: CCGAACAGGGATCTTGAACC), while cytoplasmic fractions were normalized to mitochondrial cytochrome b (For: CTAGCAGGTGTCTCCTCTATCT and Rev: GGCGTTTGGTATTGGGTTATG). Primers used for TDP43 were (For: GGGAAATCTGGTGTATGTTGTCA and Rev: TTTTCTGGACTGCTCTTTTCACT) and FUS (For: ATGGCCTCAAACGATTATACCCA and Rev: GTAACTCTGCTGTCCGTAGGG).

#### Antibodies

Antibodies used throughout the paper include Matrin 3 ab151714 and ab70336 (abcam) and HPA036565 (Sigma), Flag F3165 (Sigma) and 2368 (Cell Signaling), actin MAB1501 (Millipore), Aly ab6141 and ab202894 (abcam), ddx39b 14798–1-AP (Proteintech) and NBP2–52456 (Novus Biologicals), Sarnp HPA030902 (Sigma), and GAPDH 2118 S (Cell Signaling).

### Tissue Samples

ALS and disease control post-mortem tissue samples were obtained from the Barrow Neurological Institute ALS Tissue Bank, and the Target ALS Human Postmortem Tissue Core. All tissues samples were collected after informed consent from the subjects or by the subjects’ next of kin, complying with all relevant ethical regulations. The protocol and consent process were approved by the the Dignity Health Institutional Review Board. Clinical diagnoses were made by board certified neuropathologists according to consensus criteria for ALS. Patient demographics can be found in Supplemental Table 2.

### Data Availability

The mass spectrometry proteomics data have been deposited to the ProteomeXchange Consortium via the PRIDE partner repository with the dataset identifier PXD007710 and 10.6019/PXD007710^57,58^.

## Electronic supplementary material

Supplementary Info
