# Supplementary material for: ALS Associated Mutations in Matrin 3 Alter Protein-Protein Interactions and Impede mRNA Nuclear Export
Source: Sci Rep. 2017 Nov 6;7:14529. doi: 10.1038/s41598-017-14924-6 (PMC5674072; doi:10.1038/s41598-017-14924-6)
Supplement: Supplementary file 1 — Supplementary Info [file 41598_2017_14924_MOESM1_ESM.pdf]

ALS Associated Mutations in Matrin 3 Alter Protein-Protein Interactions and Impede mRNA Nuclear Export

Authors: Ashley Boehringer[1,2], Krystine Garcia-Mansfield[3], Gurkaran Singh[1,2], Nadine Bakkar[1], Patrick Pirrotte[3,4], Robert Bowser[1,4]*

Affiliations:

[1]Department of Neurobiology, Barrow Neurological Institute, Phoenix, AZ, USA.

[2]School of Life Sciences, Arizona State University, Phoenix, AZ, USA.

[3]Collaborative Center for Translational Mass Spectrometry, Translational Genomics Research Institute, Phoenix, AZ, USA

[4]These authors jointly directed this work

*To whom correspondence should be addressed: Robert Bowser, Department of Neurobiology, Barrow Neurological Institute, 350 W Thomas Road, Phoenix, AZ 85213. Email: Robert.bowser@dignityhealth.org.

Supplemental Table 1:

Accession	Gene	Wild Type			Ser85Cys Mutant			Phe115Cys Mutant			Pro154Ser Mutant			Thr622Ala Mutant		
		AvgP	SAINT	Manual	AvgP	SAINT	Manual	AvgP	SAINT	Manual	AvgP	SAINT	Manual	AvgP	SAINT	Manual
Q60668	Hnrnpd		+ + +	+ + +	1	+ + +	+ + +	1	+ + +	+ + +	1	+ + +	+ + +	1	+ + +	+ + +
Q61029-3	Tmpo	1	+ +	+ + +	1	+ + +	+ + +			+	0.96	+ +	+ + +			
P62270	Rps18	1	+ + +	+ + +				1	+ + +	+ + +	1	+ +	+ + +	1	+ +	+ + +
P62301	Rps13	1	+ + +	+ + +					+ +	+ + +	0.98	+ +	+ + +	0.99	+ +	+ + +
P84228	Hist1h3b	0.99	+ +	+ + +				0.96	+ +	+ + +						
O08583	Alyref	0.98	+	+												
Q9D1J3	Sarnp	0.97	+ +	+ + +		+	+ + +		+ +	+ + +	1	+ +	+ + +	0.98	+ +	+ + +
Q9QXS1-7	Plec	0.97	+ +	+ + +		+ +	+ + +									+ + +
Q9D554	Sf3a3	0.87	+	+								+				
P57784	Snrpa1	0.85	+	+					+	+		+				
Q8CH18	Ccar1	0.84	+	+	0.74	+	+	0.88	+	+		+			+	
P62281	Rps11	0.73	+	+	0.96	+ +	+	0.9	+ +	+		+	+	0.87	+	+
P08775	Polr2a	0.71	+	+					+	+	0.74	+	+		+	+
Q8BG05	Hnrnpa3			+	1	+	+	1	+	+			+			+
P05213	Tuba1b				1	+ + +	+ + +								+ +	+ + +
P99024	Tubb5				1	+ + +	+ + +									
Q91YR7	Prpf6				0.96	+	+		+		0.87	+	+			
Q9Z1N5	Ddx39b			+	0.95	+	+		+	+		+	+			+
O35691	Pnn				0.9	+	+									
Q9D0E1	Hnrnpm				0.88	+	+						+			
Q99M28	Rnps1		+	+	0.85	+	+		+	+		+	+	0.82	+	+
P14873	Map1b				0.73	+	+		+	+	0.72	+	+	0.98	+	+
Q6NZF1	Zc3h11a		+ +	+ + +				1	+ +	+ + +	1	+ + +	+ + +	1	+ + +	+ + +
P61358	Rpl27		+	+ + +				0.98	+ +	+ + +		+ +	+ + +	0.96	+ +	+ + +
P68134	Acta1							0.98	+ +	+ + +						
A2BDX3	Mocs3					+ +	+ + +	0.98	+ +	+ + +		+ +	+ + +			
P62751	Rpl23a					+ +	+ + +	0.95	+ +	+ + +				0.96	+ +	+ + +
Q9JHJ0	Tmod3		+ +	+ + +				0.95	+ +	+ + +						
Q9DBG3	Ap2b1		+					0.9	+	+	0.86	+ +	+		+	
Q8VHZ7	Imp4		+					0.7	+					0.86	+	+
Q8BG81	Poldip3		+	+		+	+		+	+	1	+	+	0.71	+	+
Q9Z1X4-2	Ilf3										1	+ +	+ + +	1	+ + +	+ + +
Q9DB96	Ngdn								+ +	+ + +	0.98	+ +	+ + +	0.98	+ +	+ + +
P62137	Ppp1ca		+ +	+ + +					+	+ + +	0.98	+ +	+ + +		+	+ + +
Q8K4P0	Wdr33										0.98	+ +	+ + +		+	+ + +
P62911	Rpl32										0.96	+ +	+ + +	1	+ +	+ + +
P62267	Rps23								+ +	+ + +	0.96	+ +	+ + +	0.96	+ +	+ + +
A2AIV2	Kiaa1429										0.96	+ +	+ + +			
Q8BL97-4	Srsf7													1	+ + +	+ + +
P20152	Vim					+ + +	+ + +							1	+ +	+ + +
Q6P5B0	Rrp12		+	+ + +					+ +	+ + +				0.98	+ +	+ + +
Q8CGP2	Hist1h2bp													0.98	+ +	+ + +
P62754	Rps6					+	+ + +							0.98	+ +	+ + +
Q9CZU3	Skiv2l2		+	+ + +					+ +	+ + +		+ +	+ + +	0.96	+ +	+ + +
Q8BMK4	Ckap4								+	+ + +				0.96	+ +	+ + +
Q8K4L0	Ddx54								+ +	+ + +		+	+ + +	0.96	+ +	+ + +
Q07646	Mest													0.96	+ +	+ + +
P62849	Rps24								+ +	+ + +		+	+ + +	0.96	+ +	+ + +
Q8C4J7	Tbl3								+ +	+ + +				0.96	+ +	+ + +
Q501J6	Ddx17								+			+		0.94	+	+
Q8CH25	Sltm								+	+		+	+	0.86	+	+
Q921Y2	Imp3													0.75	+	
P14869	Rplp0		+						+			+	+	0.7	+	+
P02535	Krt10		+						+	+						
P56959	Fus		+													
Q8CFQ3	Aqr		+													
O35326	Srsf5		+	+		+	+		+	+		+	+		+	+
Q9WV55	Vapa		+												+	+
Q91VJ5	Pqbp1		+						+							
O70503	Hsd17b12		+			+	+		+						+	+
Q9DAW6	Prpf4		+			+										
Q8QZY9	Sf3b4		+	+		+										
Q8BTI8	Srrm2		+			+						+				
Q6NV83	U2surp		+	+		+			+	+		+	+ + +			
Q9D287	Bcas2		+	+ + +												
P43276	Hist1h1b		+	+ + +		+	+ + +									
P04104	Krt1		+	+ + +		+ +	+ + +									
Q99PL5	Rrbp1		+	+ + +		+	+ + +								+ +	+ + +
Q9CQI7	Snrpb2		+	+ + +		+	+ + +		+	+ + +						
Q9QX47	Son		+	+ + +												
Q91VR2	Atp5c1		+	+ + +											+	+ + +
Q8BMS1	Hadha		+	+ + +												
Q91YE7	Rbm5		+	+ + +		+	+ + +		+ +	+ + +		+ +	+ + +			

P41105	Rpl28		+	+ + +					+	+ + +		+	+ + +			
P62900	Rpl31		+	+ + +												
P14131	Rps16		+	+ + +					+	+ + +		+	+ + +			
Q0P678	Zc3h18		+	+ + +					+ +	+ + +						
Q9JHS9	Cwc15		+ +	+ + +		+	+ + +									
Q923D5	Wbp11		+ +	+ + +												
P0C0S6	H2afz		+ +	+ + +					+	+ + +						
Q99KG3	Rbm10		+ +	+ + +		+ +	+ + +									
P61222	Abce1														+	+ + +
Q9JIX8	Acin1					+	+									
Q9QUJ7	Acsl4					+	+		+	+					+	+
O35643	Ap1b1								+	+ + +					+	+ + +
P17427	Ap2a2								+				+			
O35841	Api5								+	+ + +		+ +	+ + +		+ +	+ + +
Q3UL36	Arglu1					+	+ + +									
Q61687	Atrx														+	+ + +
Q6PDQ2	Chd4								+	+ + +						
Q9CY57	Chtop			+ + +												
Q9EPU4	Cpsf1					+			+			+				
O35218	Cpsf2														+	
Q9QXK7	Cpsf3											+	+ + +			
P63154	Crnkl1			+												
Q60737	Csnk2a1											+				
Q9CWL8	Ctnnbl1								+	+ + +						
Q3U1J4	Ddb1					+ +	+ + +									
Q8VDW0	Ddx39a								+	+ + +		+ +	+ + +			
Q9D903	Ebna1bp2			+												
Q61701	Elavl4					+	+ + +									
Q9DBE9	Ftsj3											+				
Q922P9	Glyr1								+	+ + +					+ +	+ + +
P10922	H1f0					+	+ + +									
O09106	Hdac1								+	+ + +						
Q8C2B3-2	Hdac7					+	+ + +									
P15864	Hist1h1c					+	+ + +									
P68433	Hist1h3a								+	+ + +						
Q9Z204	Hnrnpc			+			+ +			+			+ +			+ +
O35737	Hnrnph1						+ + +			+						
P61979	Hnrnpk								+			+				
P20029	Hspa5								+	+					+	
P03975	Iap														+	+
P54071	Idh2														+	
Q5SF07	Igf2bp2					+ +	+ + +		+	+ + +						
Q60749	Khdrbs1					+ +	+ + +									
Q9Z2K1	Krt16												+ + +			
Q922U2	Krt5									+						
Q8VED5	Krt79								+							
Q61595	Ktn1														+	+ + +
Q7TNC4	Luc7l2					+ +	+ + +								+	+ + +
Q3V3R1	Mthfd1l								+ +	+ + +		+ +	+ + +		+	+ + +
P46735	Myo1b								+	+ + +						
Q99104	Myo5a								+							
P12979	Myog					+ +	+ + +									
Q9D7Z3	Nol7								+						+	
Q9D6Z1	Nop56			+												
Q8BH74	Nup107														+	
Q9Z0W3	Nup160														+	+ + +
Q99JX7	Nxf1					+	+		+	+		+			+	
Q8K010	Oplah								+ +	+ + +						
P01660	P01660								+	+ + +						
Q922V4	Plrg1											+				
Q8CFI7	Polr2b								+	+ + +						
P97760	Polr2c					+	+									
P15331	Prph					+	+									
B2RY56	Rbm25											+			+	
Q8CGC6	Rbm28												+			
Q8VH51	Rbm39					+	+		+			+				
Q9WV02	Rbmx								+	+ + +					+	+ + +
Q91VM5	Rbmxl1			+			+			+			+			+
Q9CZM2	Rpl15					+	+ + +									
P62264	Rps14								+	+ + +						
Q99PL7	Scd3														+	+ + +
Q62203	Sf3a2					+	+									
Q8VIJ6	Sfpq											+			+	+
Q7TSG5	Sh3d21								+	+ + +						
P50431	Shmt1					+										

P51881	Slc25a5			+			+									
Q3UKJ7	Smu1					+ +	+ + +									
P27048	Snrpb						+ + +									
P62315	Snrpd1								+	+ + +						
P15508	Sptb								+	+ + +						
P47758	Srprb														+ +	+ + +
Q9D0B0	Srsf9									+						
Q08943	Ssrp1					+ +	+ + +		+ +	+ + +						
Q9WTS6	Tenm3					+	+ + +									
Q9ERA6	Tfip11								+	+						
B1AZI6	Thoc2								+	+ + +					+	+ + +
Q62318	Trim28			+												
P26369	U2af2					+ +	+ + +		+ +	+ + +		+	+ + +			
Q6EJB6	Utp14b											+	+ + +			
Q5SSI6	Utp18								+						+	+
Q9JI13	Utp3								+							
Q9DCD2	Xab2											+				
Q8BJ05	Zc3h14					+	+ + +									
O88532	Zfr														+	+ + +

Supplemental Table 1: High and medium confidence protein interactors of Matrin-3 wild-type, Ser85Cys mutant, Phe115Cys mutant, Pro154Ser mutant and Thr622Ala mutant. Plus signs signify a fold change over empty vector of ≤10 (+), 10-50 (+ +) or ≥50 (+ + +). Medium confidence protein interactors were defined as those identified in two out of three replicates and with a fold change ≥2.5 over empty vector in the manual analysis, or a fold change ≥2.5 over empty vector and an avgP≥0 in the SAINTexpress analysis. High confidence interactors were those with a fold change ≥2.5 over empty vector in both the SAINTexpress analysis and the manual analysis along with a SAINTexpress avgP value ≥0.7.

Supplemental Table 2:

Case ID	Gender	Age	PMI (hrs)
CON1	F	74	3
CON2	M	81	17
CON3	F	57	32
ALS1	M	72	3
ALS2	M	39	5.5
ALS3	M	83	21

Supplemental Table 2: Patient Demographics of lumbar spinal cord tissues used in the study.. PMI = post-mortem interval;. All diagnoses were performed by licensed neuropathologists.

Supplemental Figure 1

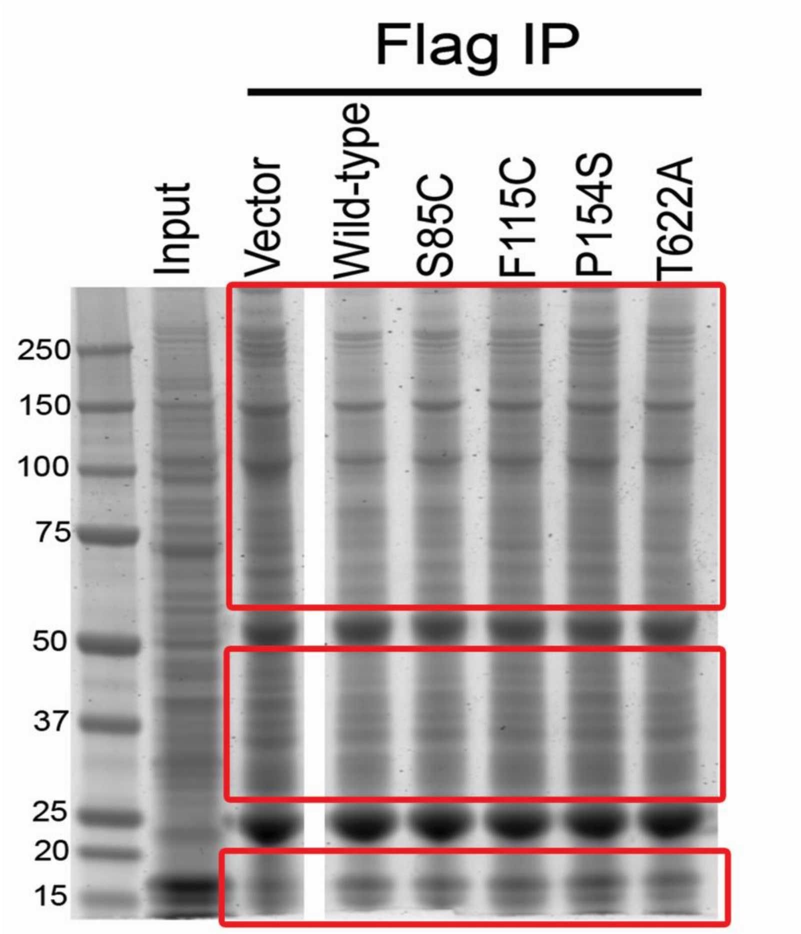

SupplementalFigure 1: Representative image of Coomassie stained gel after IP pull-down. Input indicates total nuclear lysate before immunoprecipitation experiment (40µg of total protein) followed by immunoprecipitation using Flag agarose gel in NSC-34 cells stably expressing empty vector, wild-type Matrin 3 or one of the four mutations in Matrin 3. Red boxes indicate the area used for mass spectrometry experiments (IgG heavy and light chains were removed).

Supplemental Figure 2

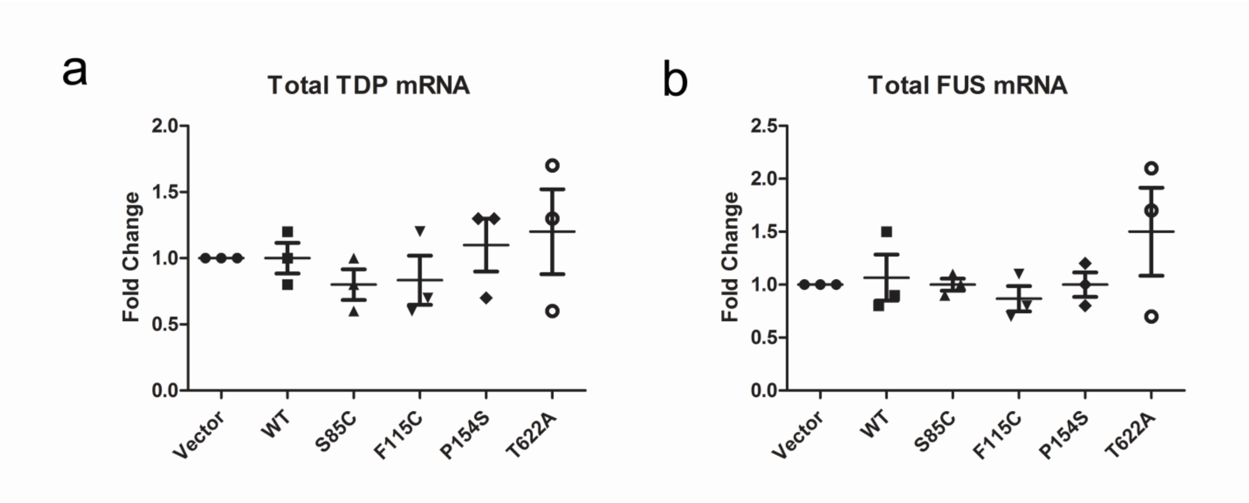

Supplemental Figure 2: Total TDP-43 and FUS mRNA levels by RT-PCR. Prior to fractionation experiments an aliquot of cells was separated and total RNA was extracted, followed by RT-PCR to determine the total levels of a) TDP-43 and b) FUS. mRNA levels were not altered by expression of wild-type or mutant Matrin 3. Error bars represent the mean +/- SEM of three independent experiments.

Supplemental Figure 3

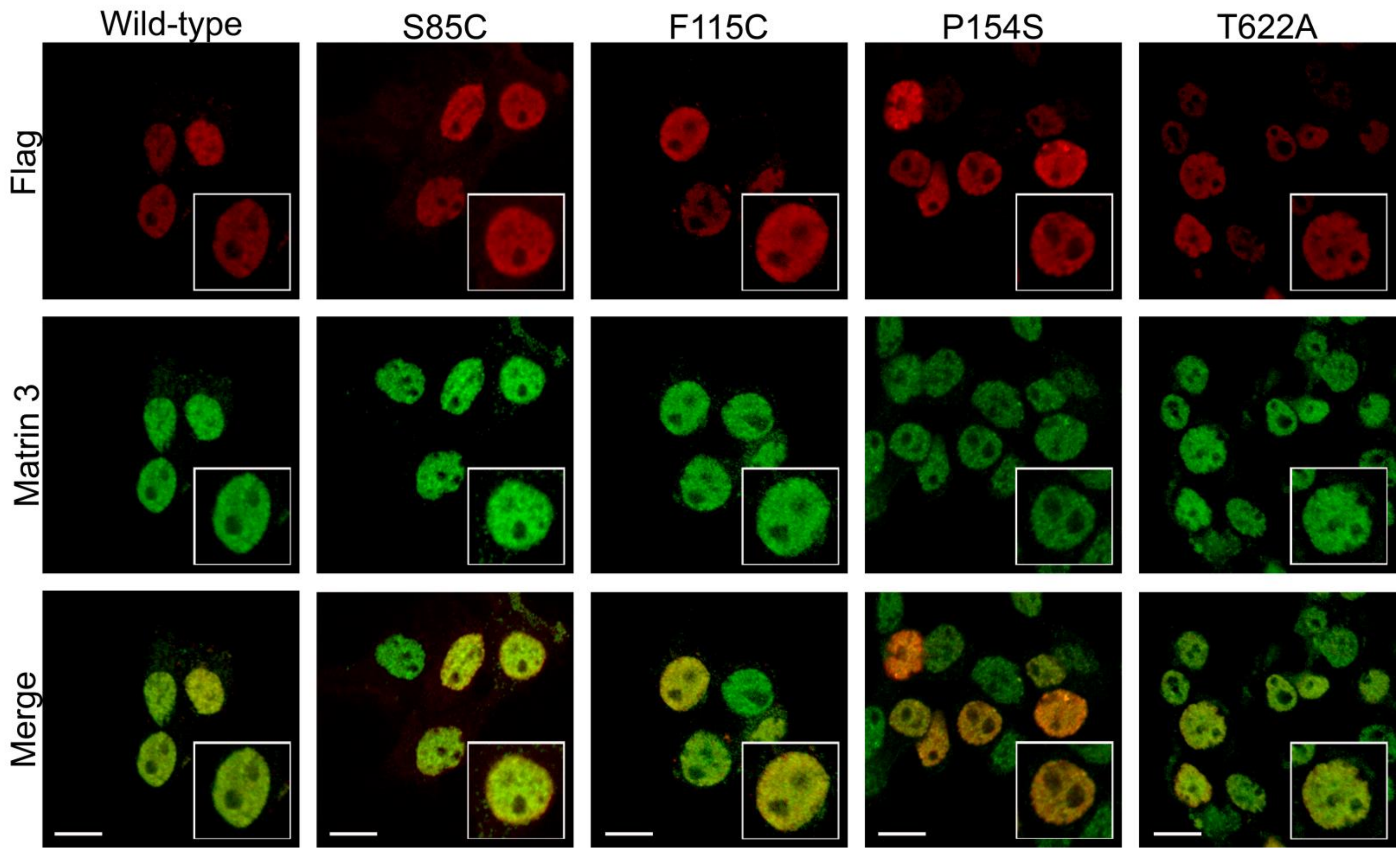

Supplemental Figure 3: Immunofluorescence images of NSC-34 cells transiently transfected with wild-type or mutant Matrin 3 then subjected to co-localization analysis. Representative images from immunofluorescence staining., flag is shown in red marking exogenous Matrin 3 and endogenous Matrin 3 is shown in green, merged image of two signals below. Insets indicate higher magnification images. Scale bar indicates 10µm.

Supplemental Figure 4

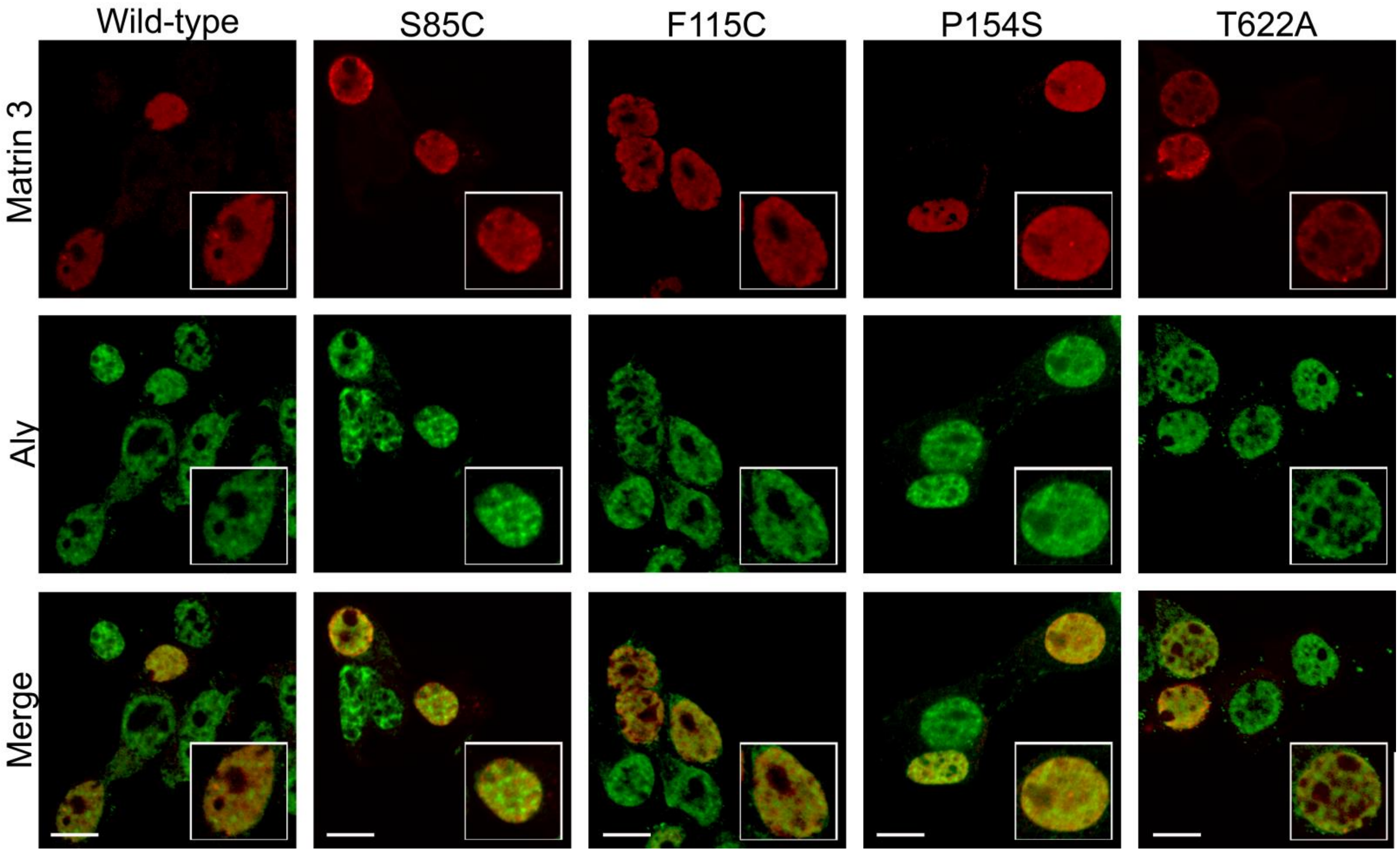

Supplemental Figure 4: Immunofluorescence images of NSC-34 cells transiently transfected with wild-type or mutant Matrin 3 then subjected to co-localization analysis. Representative images from immunofluorescence staining., flag is shown in red marking exogenous Matrin 3 and Aly is shown in green, merged image of two signals below. Insets indicate higher magnification images. Scale bar indicates 10µm.

Supplemental Figure 5

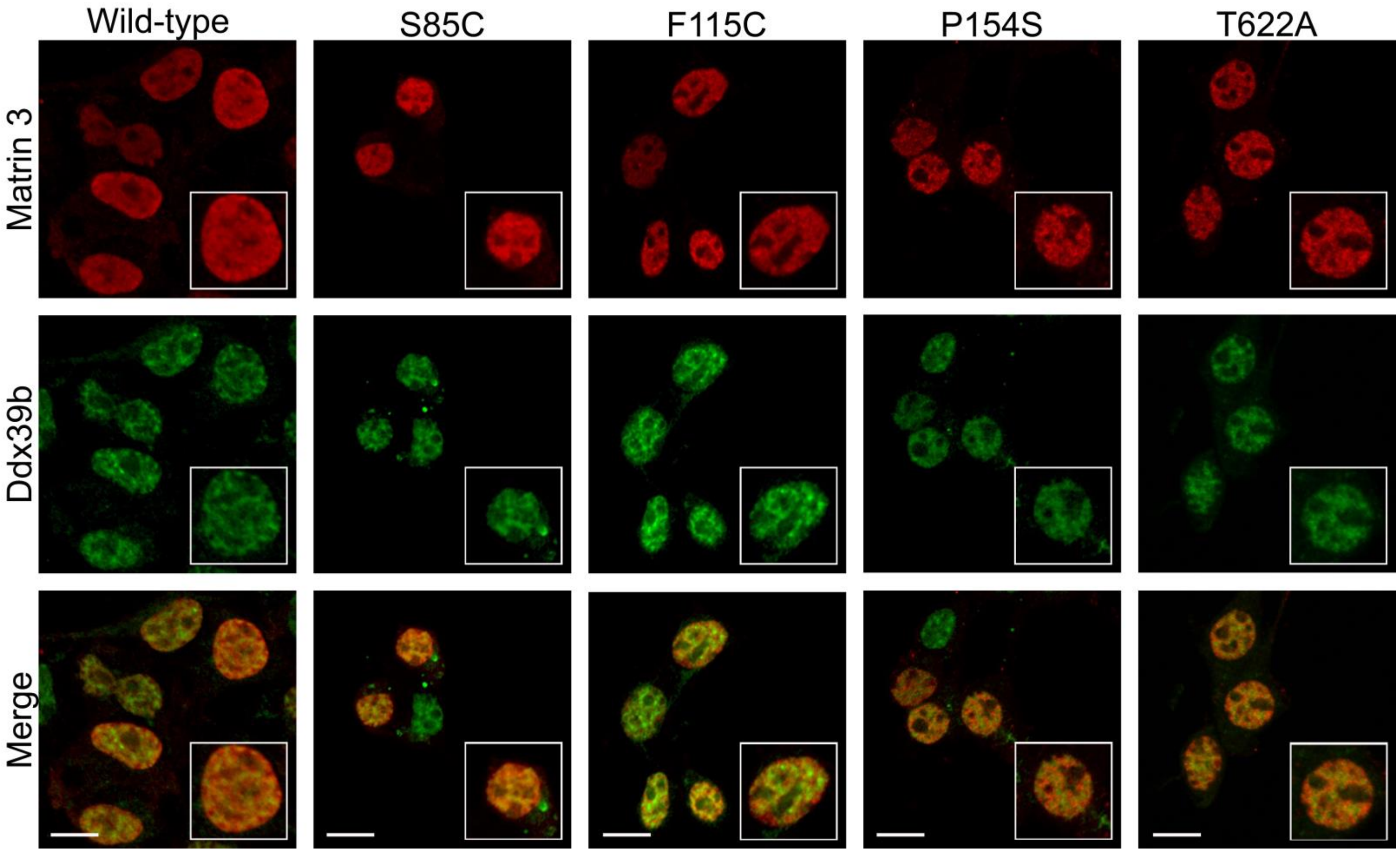

Supplemental Figure 5: Immunofluorescence images of NSC-34 cells transiently transfected with wild-type or mutant Matrin 3 then subjected to co-localization analysis. Representative images from immunofluorescence staining., flag is shown in red marking exogenous Matrin 3 and Ddx39b is shown in green, merged image of two signals below. Insets indicate higher magnification images. Scale bar indicates 10µm.

Supplemental Figure 6

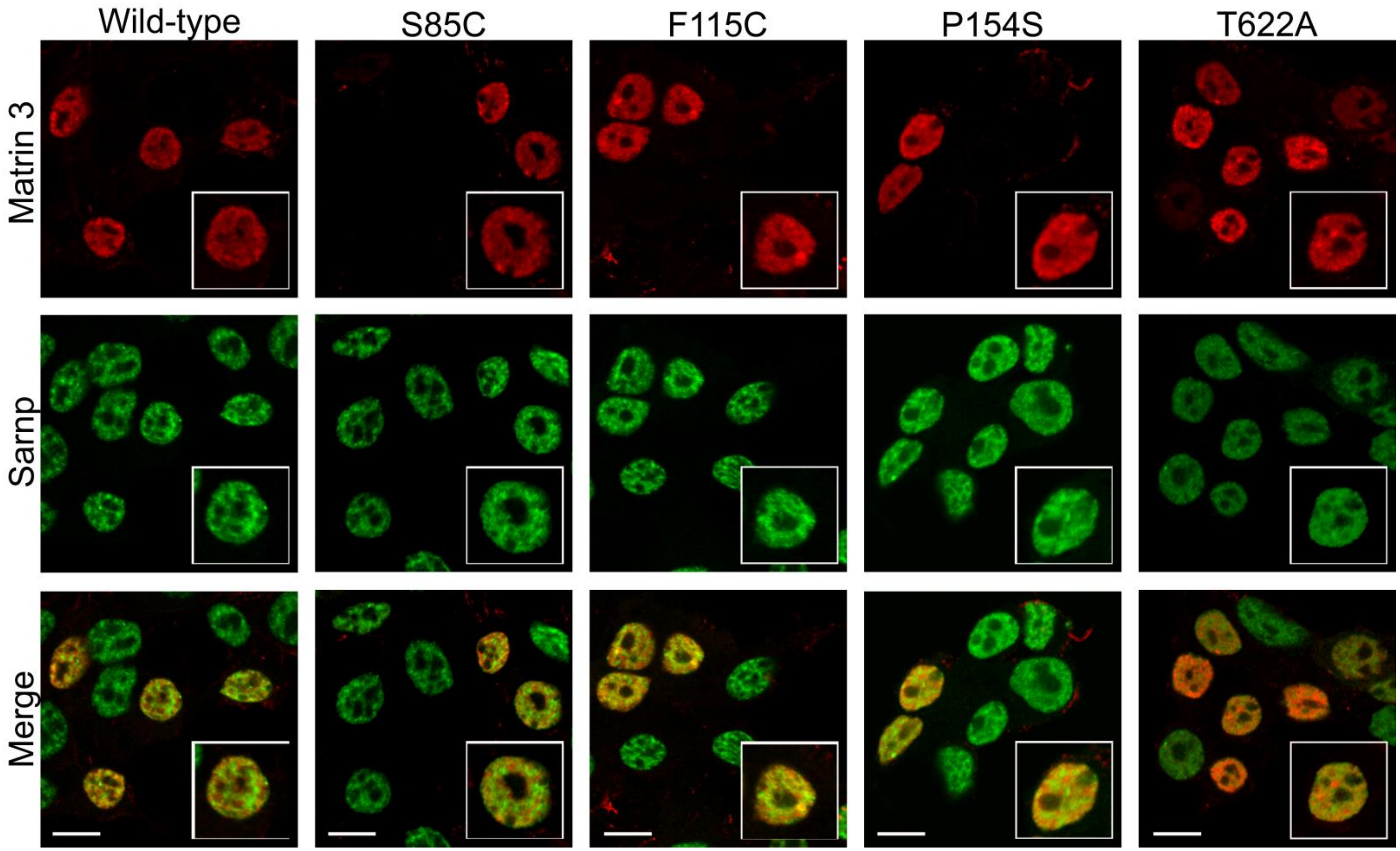

Supplemental Figure 6: Immunofluorescence images of NSC-34 cells transiently transfected with wild-type or mutant Matrin 3 then subjected to co-localization analysis. Representative images from immunofluorescence staining., flag is shown in red marking exogenous Matrin 3 and Sarnp is shown in green, merged image of two signals below. Insets indicate higher magnification images. Scale bar indicates 10μm.

Supplementary Figure 7

Figure 1c:

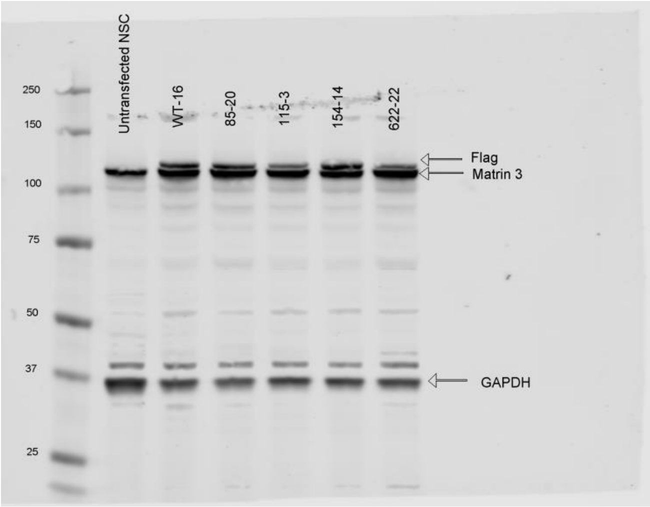

Figure 4a:

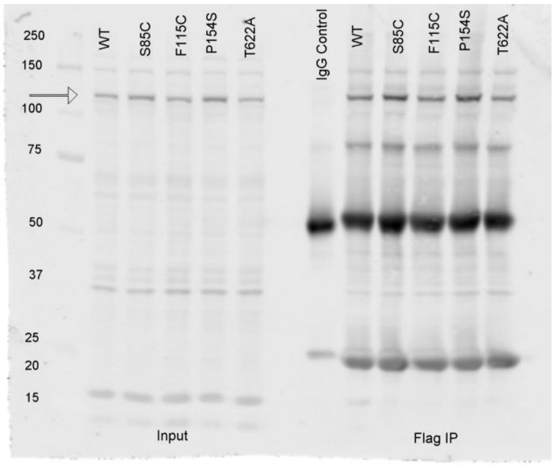

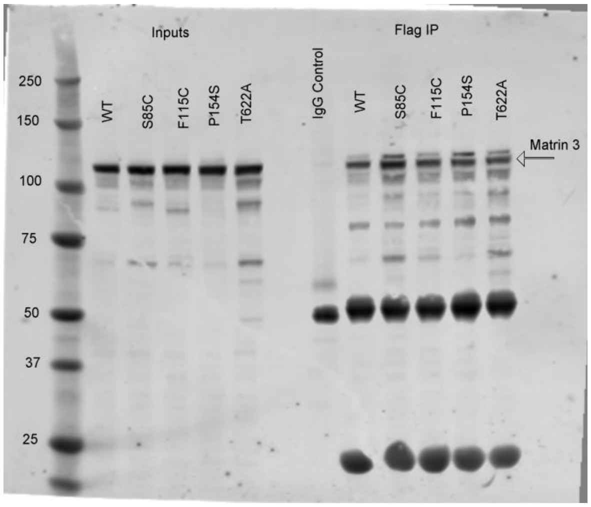

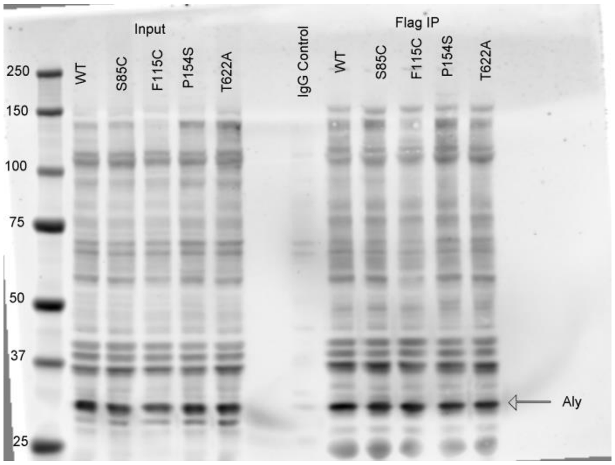

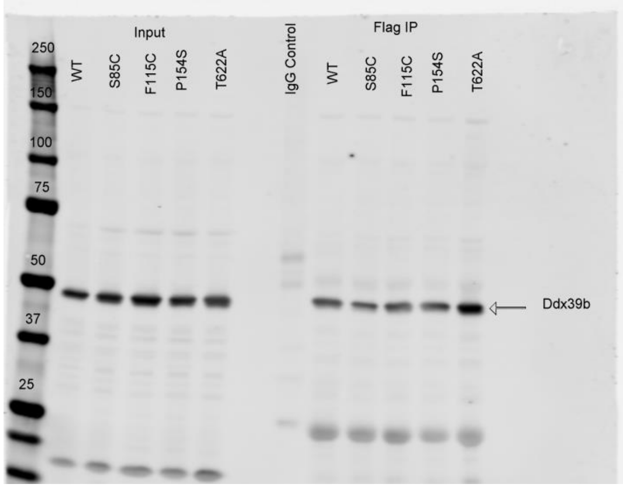

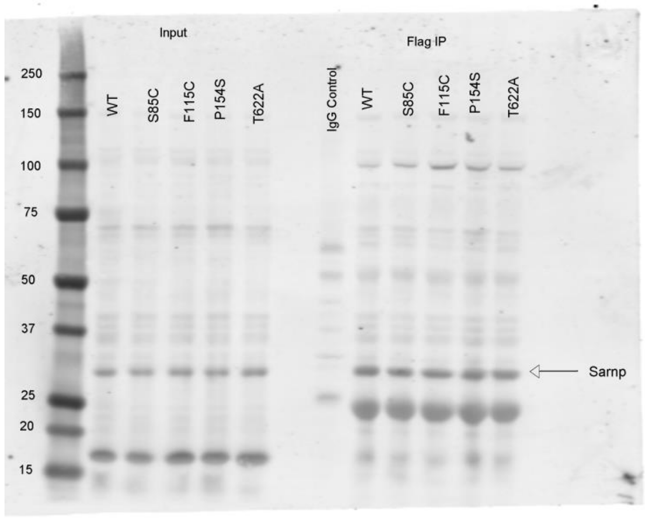

Figure 4b:

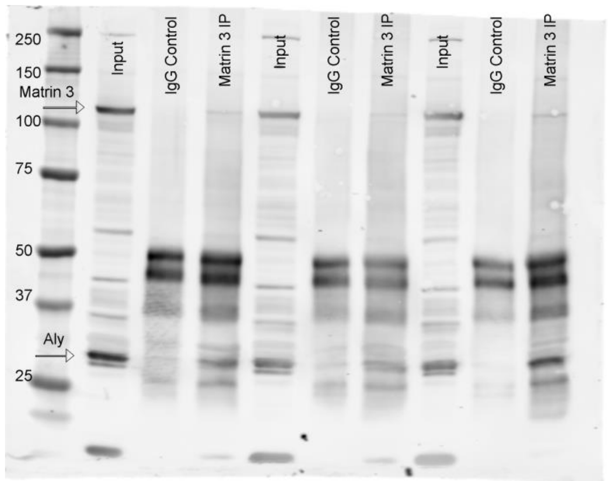

Figure 4c:

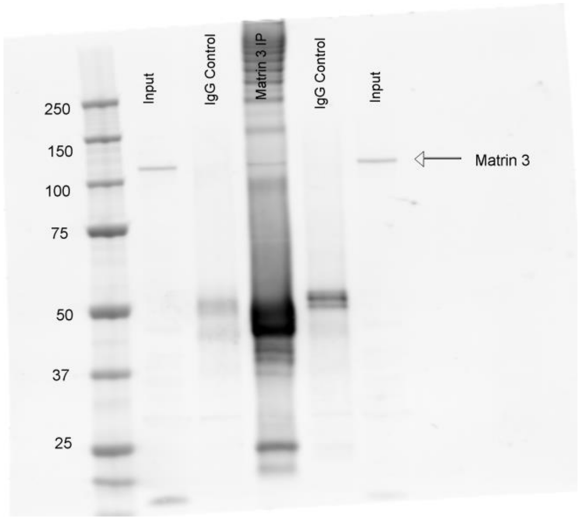

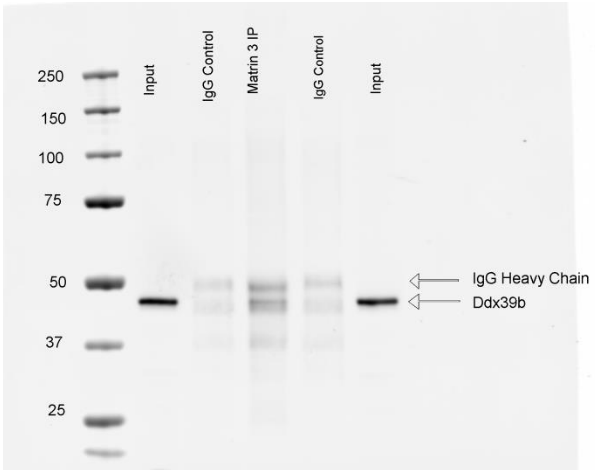

Figure 4d:

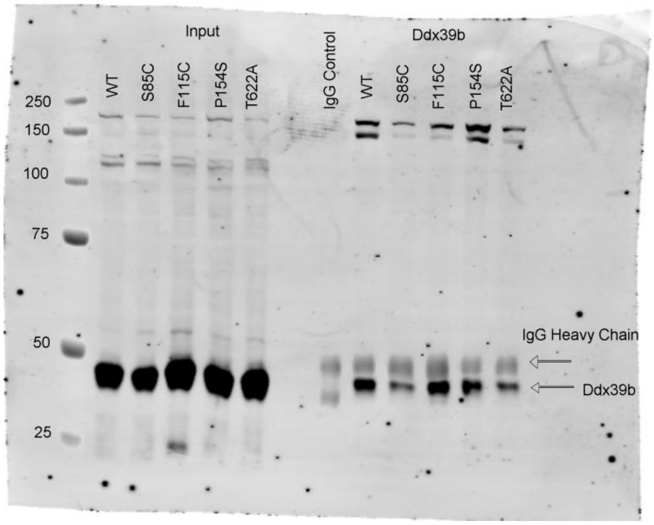

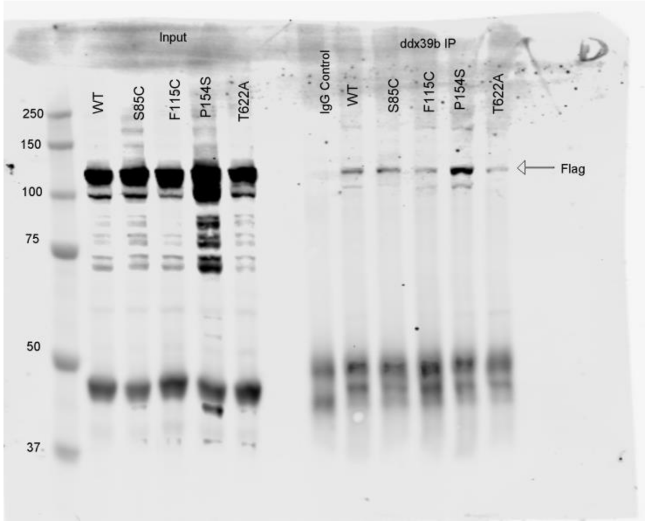

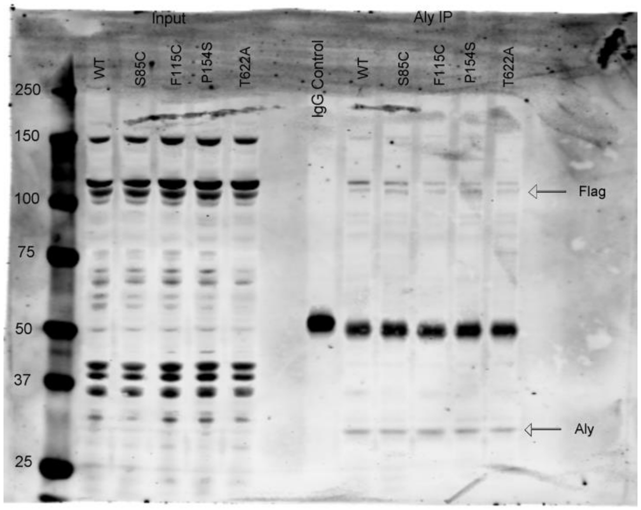

Figure 4g:

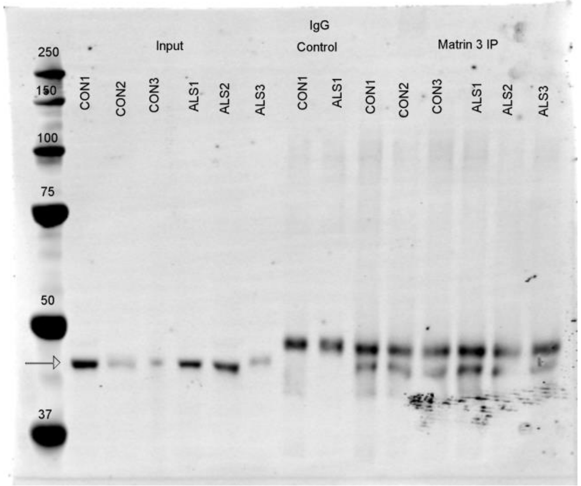

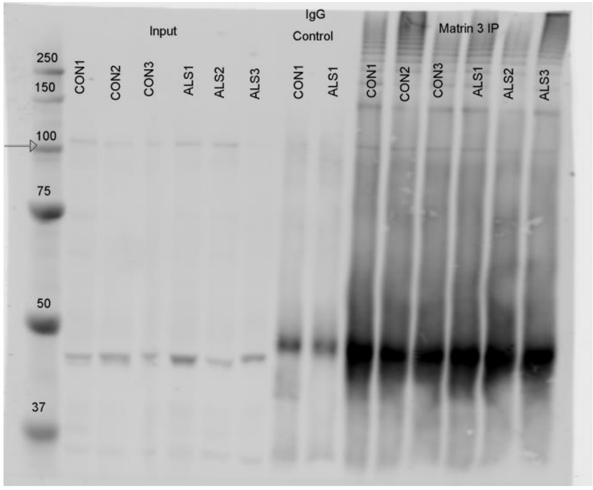

Supplementary Figure 7: Full length uncropped western blots shown in Figures 1c and 4a, 4b, 4c, 4d, 4g.
